# Low-Energy Ti Ion Irradiation-Induced Modifications in CuO Nanowires: Experimental Measurements and Computational Simulations with a 3D Monte Carlo Code

**DOI:** 10.3390/ma19204289

**Published:** 2026-10-11

**Authors:** Jingjing Yu, Yumeng Hu, Bin Liao, Xu Zhang

**Affiliations:** 1 College of Physics Science and Technology, Shenyang Normal University, Shenyang 110034, China; 2 School of Physics and Astronomy, Beijing Normal University, Beijing 100875, China

**Keywords:** ion irradiation, CuO nanowires, thermal oxidation, surface modification, Iradina, superhydrophobic

## Abstract

Ion irradiation is a unique approach for modulating the morphology and properties of nanowires (NWs). However, the ion-solid interaction and the microstructural evolution of NWs during ion beam processing remain to be further elucidated. In this work, CuO NW arrays were synthesized by thermal oxidation of copper meshes in an oxygen atmosphere and subsequently irradiated with 80 keV Ti ions at fluences ranging from 1 × 10^15^ to 5 × 10^16^ ions/cm^2^. Iradina simulations were employed to clarify the transport process of energetic ions through the NW during irradiation. Scanning electron microscope (SEM) revealed significant morphological alterations after irradiation, including bending and surface roughening. Transmission electron microscopy (TEM) provided a closer examination of individual irradiated NWs, revealing the formation of an amorphous shell on the NW surface. X-ray Photoelectron Spectroscopy (XPS) confirmed that irradiation induced the partial reduction of Cu^2+^ to lower oxidation states. Furthermore, the irradiated NW arrays exhibited enhanced hydrophobicity, achieving superhydrophobic surfaces with a water contact angle of 155 ± 1.8° at a fluence of 1 × 10^16^ ions/cm^2^. The underlying mechanisms behind these surface modifications were systematically analyzed.

## 1. Introduction

In recent years, CuO has attracted extensive attention in both fundamental research and practical applications owing to its superior characteristics, including its p-type semiconducting nature with a narrow band gap (1.2~2.1 eV) [1], high theoretical capacity (674 mAh/g) [2], low work function (2.5~2.8 eV) [3], giant magneto resistance [4], and high-temperature superconductors [5]. Compared with its micro- or bulk counterparts, CuO nanostructures exhibit fascinating physical and chemical properties derived from the small-size effect, surface effect, quantum confinement effect, and Coulomb blockade effect [5]. These nanostructures have been widely studied for promising applications in catalysis [6], lithium-ion batteries [7], solar cells [8], gas sensors [9], field emission emitters [10], hydrogen storage [11], oil-water separation [12], fog collection [13], and many others [14,15]. However, practical applications increasingly demand CuO nanostructures with enhanced performance. Therefore, the rational design and modification of CuO nanostructures have become a highly active research topic. Numerous physical and chemical methods have been developed to implement a wide range of modification strategies, such as element doping [14], defect engineering [16], coupling of multiple materials [17,18,19], and micro/nanostructural design [20].

Among these approaches, ion beam technology, including ion implantation and ion irradiation, is regarded as an efficient and powerful tool for modifying CuO nanostructures [21,22,23,24,25]. This technology is based on the Coulomb electromagnetic interaction between energetic ions and individual atoms within the target material [26]. During collisions between incident ions and the target, energetic ions transfer energy to either the nuclei or the electrons of target atoms, leading to various atomistic effects such as doping, atomic displacement, and sputtering. These effects substantially influence the electronic and atomic structures of the target material, thereby enabling material modification. As a pure physical process that does not involve chemical solvents or reagents, ion beam technology offers high atomic economy and avoids serious environmental issues. More importantly, it exhibits superior controllability, accuracy, and repeatability [27]. Ion implantation is broadly employed to introduce dopants into target materials to tailor their electronic structures or to fabricate a range of nanostructures [28]. Compared with doping during growth and diffusion, ion implantation doping is not limited by the solid solubility of elements in materials and can achieve forced and precise doping of almost all elements without introducing other impurity elements [29]. By comparison, ion irradiation is a promising technology for engineering defects in target materials, enabling facile modulation of morphologies and properties [26,27,28,29].

To date, the main achievements in utilizing ion beam technology to modify CuO nanostructures are as follows. Bind et al. observed phase transformations and increased electrical conductivity in CuO films following low-energy nitrogen ion implantation [21]. Shi et al. discovered that argon ion irradiation induces room-temperature ferromagnetic properties in CuO thin films [22]. Song et al. examined the amorphization behavior and enhanced field emission characteristics of CuO NWs after argon ion beam exposure [23]. Sisman et al. demonstrated that the gas-sensing properties of CuO NWs are markedly improved through iron and nitrogen ion irradiation [24]. Chaudhary et al. investigated the effects of gold ion irradiation on the photoelectrochemical and structural properties of CuO thin films [25]. Although many achievements have been made over the past few decades, most publications primarily concentrate on experimental measurements aimed at establishing correlations between the microstructures and properties of CuO nanostructures. However, the fundamental mechanisms underlying the modifications of CuO nanostructures are still not fully understood. Investigating these mechanisms solely through experimental approaches poses considerable challenges. Therefore, it is essential to conduct targeted theoretical simulations in conjunction with experimental studies to gain a comprehensive understanding of these modifications. So far, relatively little information is available on theoretical studies regarding the interaction between ion beams and CuO nanostructures.

Theoretical studies on ion-solid interaction have been advanced by the development of computer simulation codes, primarily based on Monte Carlo (MC) simulations of binary collision processes [30,31,32,33,34]. Among these, SRIM is the most widely used due to its user-friendly interface, extensive database on compound targets, and electronic energy loss data [34]. However, SRIM defines the target as a stack of sharp-interfaced layers with infinite lateral extension, such as thin films and multilayers [30]. Consequently, when applied to nanostructures, the results sacrifice accuracy, because the effects of the same ion beam on bulk materials and nanostructures differ considerably [34,35]: (i) since the geometrical dimensions of nanostructures are comparable to the ion range, incident ions may escape laterally without contributing to doping; (ii) the number of collision cascades is significantly reduced due to the shortened interaction distance; and (iii) the anisotropic surface geometry of nanostructures leads to a higher sputter ratio caused by a locally ion impact angle. Therefore, SRIM is not ideally suited for simulating ion beam interactions with nanostructures.

Several other MC codes, including Iradina, TRI3DYN, and IM3D, are capable of incorporating three-dimensional (3D) target structures in the input file [36]. Of these, only Iradina is truly open-source and freely available, offering an alternative to SRIM for simulating ion beam irradiation/implantation of nanostructures [36,37]. Originally developed by Borschel and Ronning at the University of Jena [33], Iradina models geometrically complex nanostructures using a rectangular box of arbitrary size, which can be subdivided into equal-sized rectangular cells whose dimensions can be reduced to the nanometer scale to capture the curved surface geometry of nanowires or nanoparticles [31]. It is written in standard C, runs on most common computers and operating systems, employs look-up functions from the Corteo databases for fast computation, and provides a user-friendly interface suitable for researchers not specialized in numerical simulations [36,37,38]. To date, many research groups have reported studies on ion irradiation/implantation in nanostructures using Iradina, demonstrating more realistic results compared with other simulation codes [31,33,35,38,39,40].

To the best of our knowledge, no published reports have yet addressed the simulation of ion beams within CuO nanostructures using Iradina. In the present study, thermally oxidized CuO NW arrays were irradiated with an 80 keV Ti ion beam at fluences ranging from 1 × 10^15^ to 5 × 10^16^ ions/cm^2^, in order to systematically investigate variations in morphology, microstructure, surface chemical composition, and wettability. Theoretical calculations were performed using the Iradina code to simulate the interaction processes between the ion beam and the NWs. The corresponding mechanisms accounting for the observed phenomena were discussed in details.

## 2. Experimental Method

### 2.1. Materials

High-purity (99.99%) Cu mesh was purchased from Shanghai Hua-Xin Hardware Product Co., Ltd. (Shanghai, China). Acetone, hydrochloric acid (HCl, 36–38%), and ethanol absolute (≥99.7%) were supplied by Beijing Chemical Works (Beijing, China). Deionized water (DI water) was prepared in the laboratory.

### 2.2. Thermal Oxidation Synthesis of CuO NWs

The Cu mesh (0.15 mm thickness) was cut into pieces of about 1 cm × 1 cm. The Cu substrates were then cleaned using a 1 M HCl solution (0.5 min), thoroughly rinsed with DI water, immersed in acetone and ethanol for an ultrasonic bath (5 min each), and finally dried under a nitrogen flow. This process effectively removes impurities and native oxides from the metal surface. The cleaned substrates were placed in an aluminum boat and transferred into the center of a tube furnace. The furnace was pumped to a pressure of ~1.3 × 10^−6^ Pa, and oxygen gas was then admitted into the furnace to the pressure of ~2.7 × 10^4^ Pa. The substrates were heated up to 450 °C (10 °C/min) in oxygen gas, oxidized at that temperature for 60 min in static oxygen (10 mL/min), and then cooled down to room temperature at the same rate in oxygen. A layer of oxide with CuO NWs was formed on the top of the substrates during this process.

### 2.3. Irradiation Experiments

Ion irradiation experiments were carried out in the School of Physics and Astronomy, Beijing Normal University (Beijing, China). The oxidized samples were irradiated by high-current ion beams generated by a metal vapor vacuum arc (MEVVA) ion source with an incident angle of 90°. The chamber was evacuated to a pressure of 3.0 × 10^−3^ Pa during irradiation. The cathode ion source electrode was pure titanium (Ti, 99.9%). The extraction voltage was set to 40 kV. Metal ions extracted from the MEVVA source usually have multiple charge states. For Ti ions, the weighted average charge state was measured to be 2. Therefore, the average energy of Ti ions was calculated to be 80 keV. The ion beam density was approximately 0.5 mA and the fluences used to irradiate the samples were 1 × 10^15^, 5 × 10^15^, 1 × 10^16^ and 5 × 10^16^ ions/cm^2^.

### 2.4. Characterizations

The surface morphologies of CuO NWs on Cu substrates before and after irradiation were investigated with field-emission scanning emission microscopy (FE-SEM; Hitachi S-4800, Tokyo, Japan). The SEM images were processed using Digital Micrograph software (v3.5. https://www.gatan.com/installation-instructions (accessed on 10 March 2025)). The diameter and length of CuO NWs from SEM images were analyzed employing Nano Measurer software (v1.2.5. https://soft.china.com/details/13/2137410.html (accessed on 9 May 2025)). Microstructural analyses of the samples were performed by using a transmission electron microscopy (TEM; FEI Technai G2 F20, Hillsboro, USA). The common TEM images, including low-magnification and high-resolution transmission electron microscopy (HRTEM) images, were processed by Digital Micrograph 3.5. The selected area electron diffraction (SAED) patterns were calibrated utilizing the TEM data analytics platform from Sinoma Institute of Materials Research Co., Ltd. (Guangzhou, China). The surface chemical composition of the samples was characterized by X-ray photoelectron spectroscopy (XPS; VG ESCALAB MKII, St Leonards-on-Sea, UK) with an Al-Kα X-ray source (1486.6 eV). The binding energy for the C1s peak (284.8 eV) was used as an internal reference. Spectral analysis was performed using peak fitting software (Avantage v6.7.0. http://www.surfsciftp.co.uk/avant5/download.php (accessed on 6 April 2025)) after Linear background subtraction. The static contact angle (CA) of the samples with 5 μL water droplets was measured with a contact angle meter (POWEREACH, JC2000D1, Shanghai, China) at room temperature. Five-point fitting method was used to measure the CA.

### 2.5. Computational Simulations

**Figure 1:**
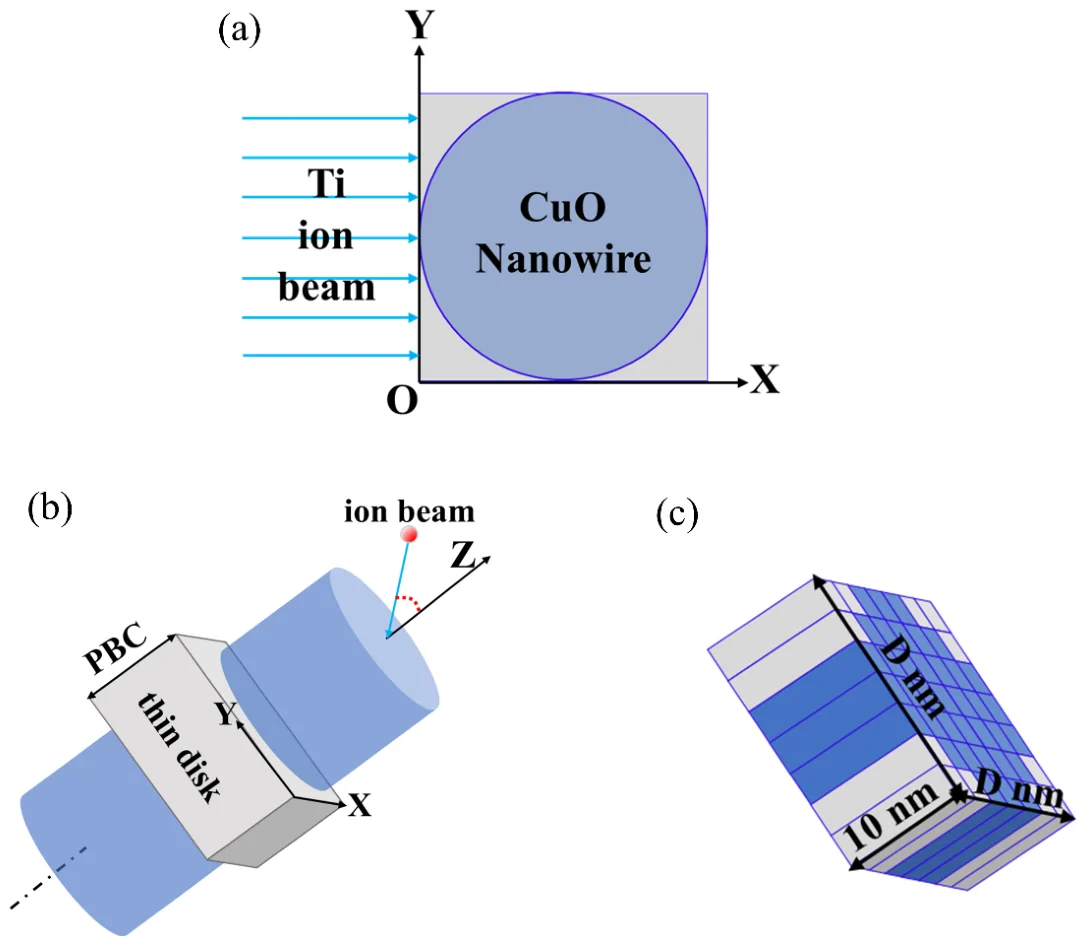
Simulation schematic of a CuO NW irradiated by Ti ions: (**a**) the ions impinge on the NW along the X-direction at random positions across the Y-axis; (**b**) schematics of the simulation geometry in Iradina; (**c**) the simulation volume is divided into equal-sized rectangular cells. The D represents the diameter of the NW.

The simulations were carried out using the nanowire version of Iradina (v1.0.8. https://sourceforge.net/projects/iradina/files/Iradina_Jena/ (accessed on 5 March 2024)). Figure 1 shows the simulation schematic of a CuO NW irradiated by Ti ions in Iradina. As demonstrated in Figure 1a, the NW was considered as a solid cylinder with a diameter of D nm. The incoming direction of the Ti ion beam was chosen to be the +X axis, perpendicular to the length of the NW. Figure 1b shows that a small segment of the NW was considered for the simulation, and a periodic boundary condition was applied along the NW length (Z-direction). Thus, the simulation implies translational symmetry along the NW axis and infinite length. Figure 1c depicts the simulation target volume of the NW, which was divided into 50 × 50 × 1 cells. Each cell had a size of (D/50) nm × (D/50) nm × 10 nm.

The cells inside the NW were filled with the target material (CuO). The cells outside the NW were set to vacuum in order to keep track of the target atoms and/or ions leaving the simulation volume for accurately calculating the sputtering of CuO. The target was irradiated by 10^6^ incident ions with random entry positions distributed over the Y-axis. The surface binding energy (Cu: 3.52 eV, O: 2 eV), lattice binding energy (Cu: 3 eV, O: 3 eV), and displacement energy (Cu: 25 eV, O: 28 eV) used in the simulation were the default values obtained from the SRIM tables. The above simulations using the Iradina code were completed on a personal computer (AMD Ryzen 7 5800X 8-Core processor, Taiwan, China). The output data were processed using Matlab (vR2022b. https://www.mathworks.com/products/new_products/release2022b.html (accessed on 26 September 2024)) and Origin (v2022. https://www.originlab.com/2022 (accessed on 4 October 2024)).

## 3. Results and Discussions

### 3.1. Morphology Characterization

**Figure 2:**
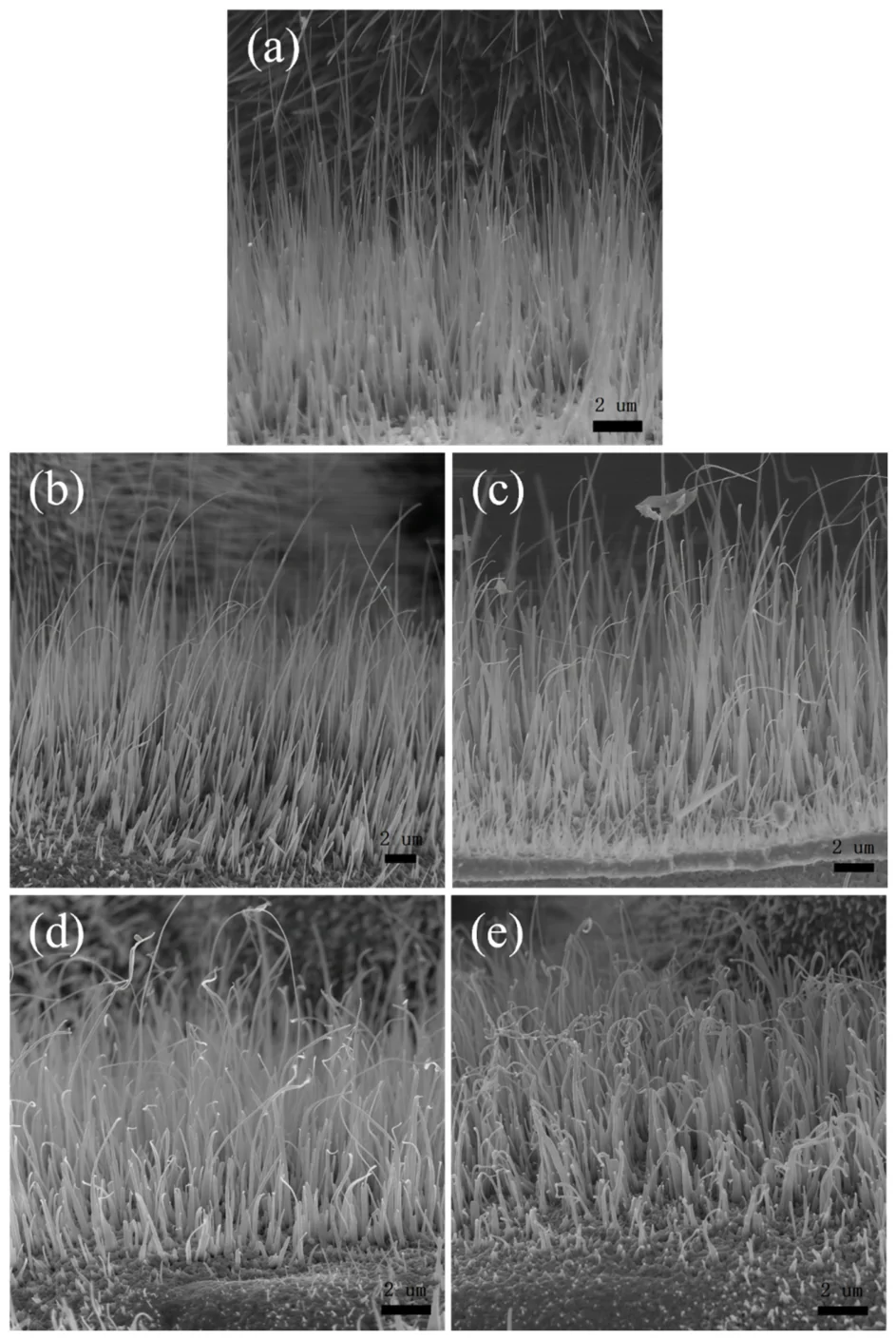
Side-view SEM images of 80 keV Ti ion irradiated CuO NWs with different fluences: (**a**) 0; (**b**) 1 × 10^15^; (**c**) 5 × 10^15^; (**d**) 1 × 10^16^; (**e**) 5 × 10^16^ ions/cm^2^.

Figure 2 shows the side-view morphologies of CuO NWs before and after Ti ion irradiation at different fluence. As shown in Figure 2a, most pristine NWs are initially straight and oriented perpendicularly to the substrate plane. The diameter at the base is slightly larger than that near the tip, and both diameter and length vary among individual NWs, ranging from 30 to 90 nm in diameter and 2 to 18 μm in length. Figure 2b shows the morphology of NWs irradiated at a fluence of 1×10^15^ ions/cm^2^, where longer and thinner NWs exhibited pronounced bending, whereas shorter and thicker counterparts show no similar deformation. As the ion fluence increases [Figure 2c–e], bending toward the substrate intensifies, accompanied by a notable reduction in NW length. At a fluence of 1 × 10^16^ ions/cm^2^ [Figure 2d], the NWs no longer bent uniformly in a single direction; instead, their tips exhibit reverse bending, resulting in an S-shaped morphology. At the maximum fluence of 5 × 10^16^ ions/cm^2^, some NWs fracture at their tips [Figure 2e]. Although the NWs vary in diameter and length, a one-to-one correlation appears to exist between bending degree/direction and the ion fluence.

**Figure 3:**
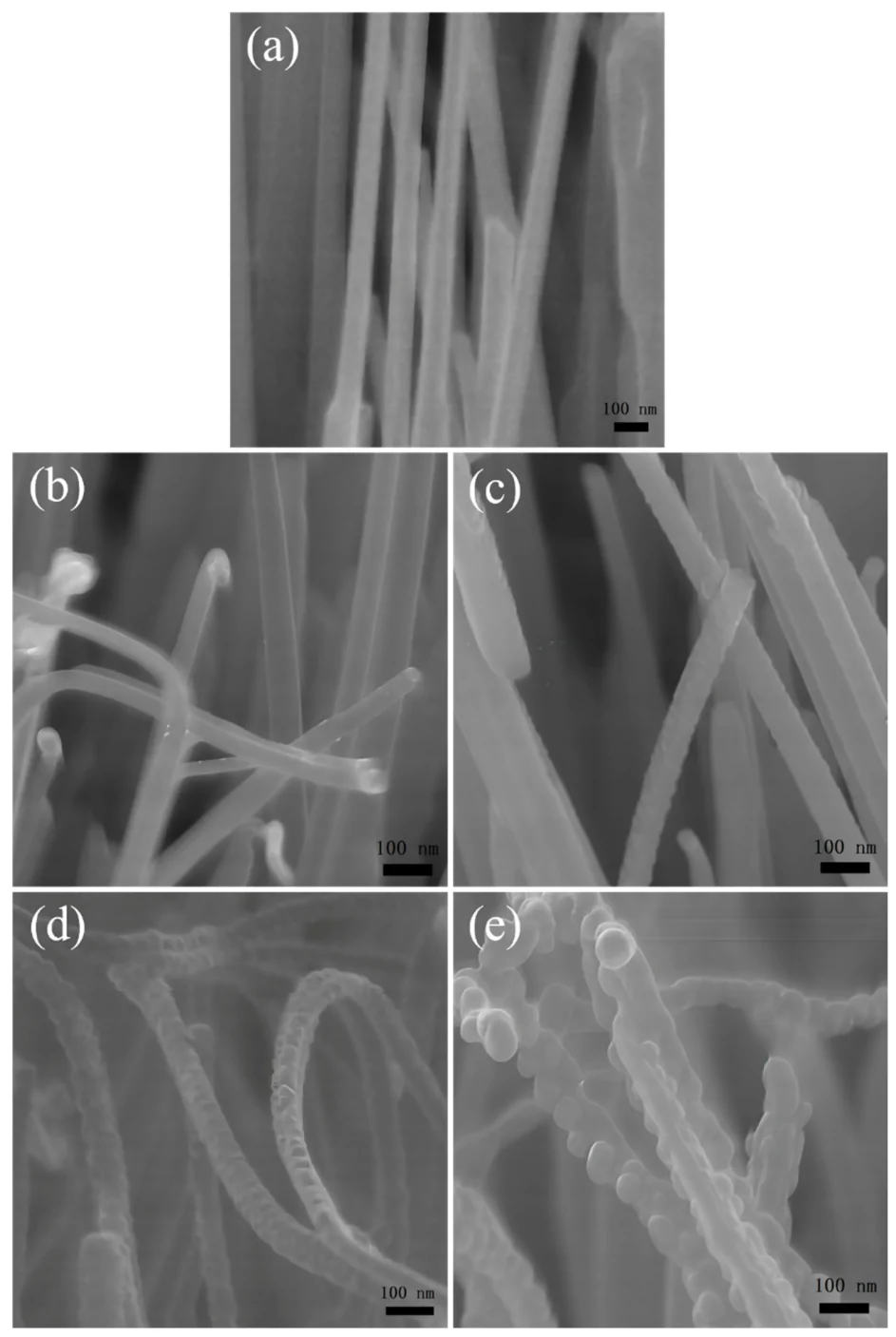
Corresponding high-magnification SEM images of 80 keV Ti ion irradiated CuO NWs with different fluences: (**a**) 0; (**b**) 1 × 10^15^; (**c**) 5 × 10^15^; (**d**) 1 × 10^16^; (**e**) 5 × 10^16^ ions/cm^2^.

Figure 3 presents the high-magnification SEM images of the NW samples, allowing detailed observation of their morphological characteristics. Figure 3a shows that the NWs exhibit very smooth surfaces prior to irradiation. At a low fluence of 1 × 10^15^ ions/cm^2^ [Figure 3b], no significant surface alterations are observed. Small node-like structures begin to appear at a moderate fluence of 5 × 10^15^ ions/cm^2^ [Figure 3c]. At higher fluences [Figure 3c–e], numerous protrusions emerge on the NW surfaces, and their size increased progressively with increasing ion fluence. These smaller node-like structures are likely the initial stage of protrusion formation. Evidently, the formation of these protrusions contributes to the roughening of the NW surfaces.

### 3.2. Microstructure Characterization

**Figure 4:**
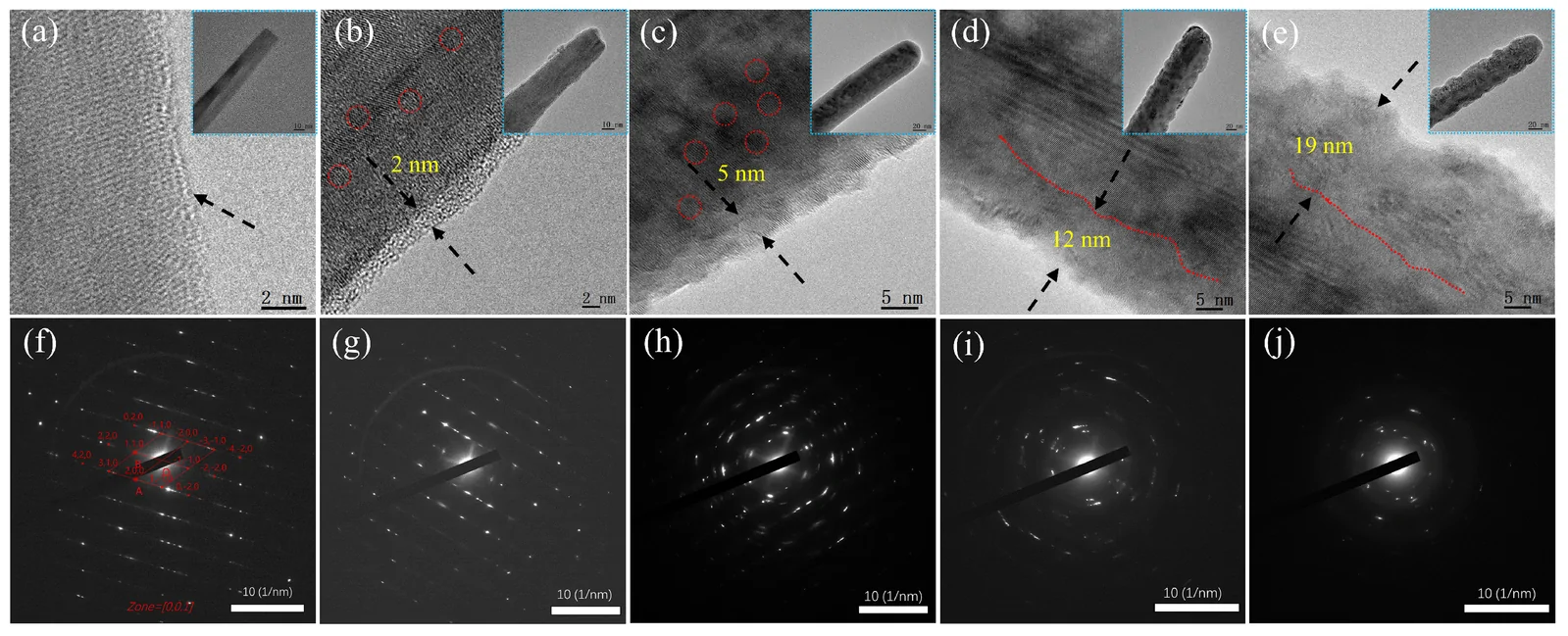
HRTEM images (**a**–**e**) and corresponding SAED patterns (**f**–**j**) of 80 keV Ti ion irradiated CuO NWs with different fluences: (**a**,**f**) 0; (**b**,**g**) 1 × 10^15^; (**c**,**h**) 5 × 10^15^; (**d**,**i**) 1 × 10^16^; (**e**,**j**) 5 × 10^16^ ions/cm^2^. Inserted images (**a**–**e**) show the corresponding low-magnification TEM images.

The microstructural evolution of CuO NWs induced by Ti ion irradiation was investigated in detail using TEM and HRTEM. Figure 4 presents the HRTEM images [Figure 4a–e] and SAED patterns [Figure 4f–j] of the NW samples.

Figure 4a illustrates that the pristine NW exhibits a regular atomic arrangement, indicative of high crystallinity. The inset of Figure 4a reveals that the NW is divided by a twin boundary, where differing crystal orientations result in noticeable contrast differences in the bright-field TEM image. The corresponding SAED pattern [Figure 4f] confirms the bi-crystalline structure of the NW, consisting of two sets of diffraction spots with mirror symmetry [41]. The zone axis lies along the [001] direction of monoclinic CuO as calculated from the bright spots [Figure 4f].

Figure 4b–e demonstrate that the original crystalline orientation of the NWs is progressively lost following Ti ion irradiation. Figure 4b shows that low-fluence irradiation results in the formation of a small amount of short-range-ordered nanograins on the NW surface (indicated by red circles). As shown in Figure 4c, the number of nanograins markedly increases with ion fluence. Figure 4d,e show that high-fluence irradiation causes extensive amorphous regions. The corresponding SAED patterns [Figure 4h–j] exhibit a transition from a regular pattern to a ring pattern, indicating a transformation from a perfect crystalline structure to a polycrystalline structure and ultimately to an amorphous phase. Consequently, an amorphous shell layer forms on the surface of the irradiated NWs. Its total thickness increases from ~2 nm to ~19 nm with increasing fluence [Figure 4b–e], representing a layered accumulation of defects. Notably, the bulk crystalline zone adjacent to the amorphous layer retains a certain degree of crystallinity even at the highest fluence of 5 × 10^16^ ions/cm^2^, revealing that the threshold fluence required for complete amorphization likely exceeds 5 × 10^16^ ions/cm^2^.

Finally, Ti ion bombardment leads to the formation of sharper tips and jagged contours along the NW surface, as shown in Figure 4a–e. A detailed observation of the jagged contours at each fluence reveals that depressions are a predominant feature at lower fluences [Figure 4b,c]; these depressions expand into larger craters at higher fluences [Figure 4d,e], exhibiting the characteristic of a crater with two adjacent protrusions. The formation of craters and protrusions results in non-uniform roughening of the NW surfaces, in agreement with the SEM analysis [Figure 3c,d]. These findings indicate that ion irradiation can quantitatively introduce defects at the atomic level through precise control of ion fluence [29].

### 3.3. Characterization of Surface Composition

XPS was employed to investigate the effect of Ti ion irradiation on the surface chemical composition of CuO NWs.

**Figure 5:**
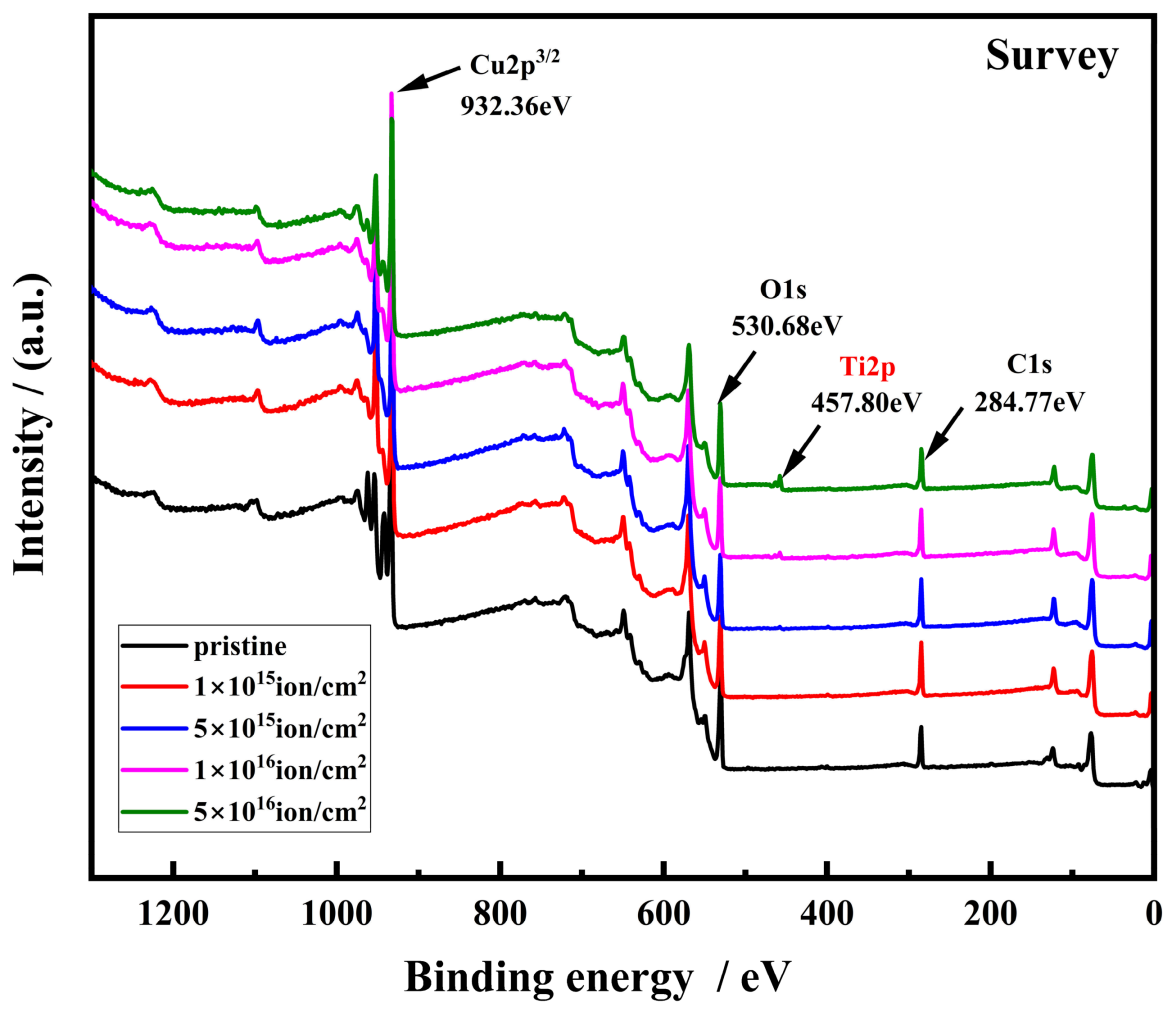
XPS core level full spectra of CuO NWs before and after Ti ion irradiation with different ion fluences.

Figure 5 presents the broad-scan survey spectra for pristine NWs and samples irradiated at different fluences. Ti, O and Cu elements are detectable in the samples. Figure S1 shows the high-resolution Ti 2p spectra for the irradiated samples. The peaks centered at 463.6 eV (2p_1/2_ orbital) and 457.9 eV (2p_3/2_ orbital) are attributed to the Ti^4+^ [42]. With increasing ion fluence, the intensity of Ti 2p peaks increases, corresponding to an increase in Ti atomic percentage in the NWs.

**Figure 6:**
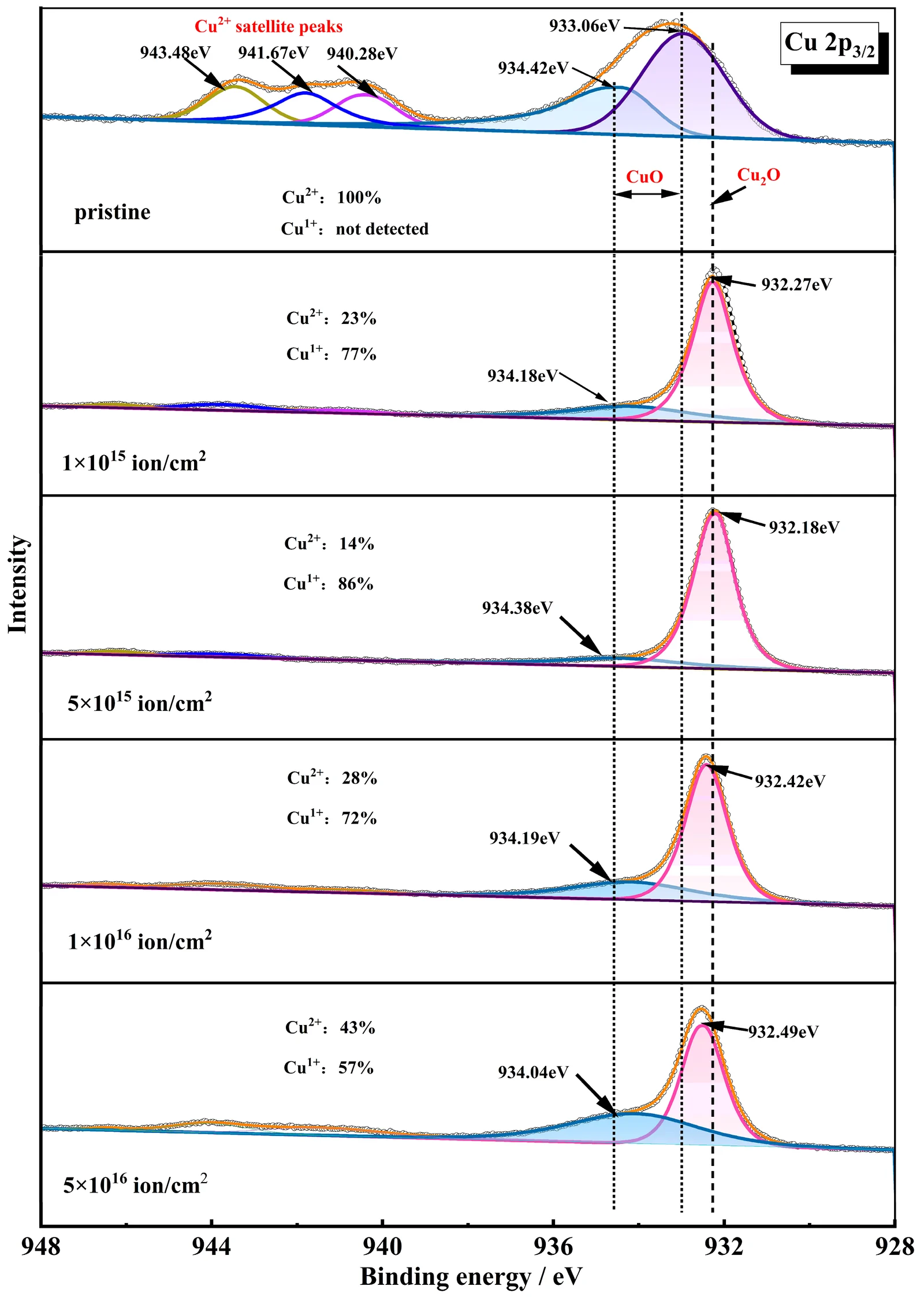
High-resolution XPS Cu2p_3/2_ spectra of CuO NWs before and after Ti ion bombardment with different ion fluences.

Figure 6 shows the high-resolution Cu2p_3/2_ spectra for the NW samples. The peak fit of the pristine NW spectrum shows characteristic features associated with the Cu^2+^ chemical state in CuO species [23]. Specifically, two main photoemission peaks centered at 933.06 eV and 934.42 eV, accompanied by three shake-up satellite peaks at 940.28 eV, 941.67 eV, and 943.48 eV. After ion irradiation, the shapes of the Cu2p_3/2_ peaks undergo significant changes. The intensities of all signals associated with Cu^2+^ decrease rapidly, and a new main peak appears at approximately 932.27 eV, typically representing Cu_2_O and/or metallic Cu species due to overlapped peaks corresponding to Cu^1+^ and Cu^0^ states [43]. This observation suggests that a fraction of CuO on the NW surface undergoes partial reduction, yielding either Cu_2_O, metallic Cu, or a mixture of both phases [24]. According to Ho et al. [44], the decomposition products generated by ion bombardment of metal oxides differ significantly from those of metal oxyanion salts: reduction to lower metal oxidation states is consistently observed in all oxyanion compounds, whereas reduction to the metallic state occurs only in some metal oxyanion salts and not in the corresponding metal oxides. Therefore, in our study, it can be inferred that the only reduction product of ion-irradiated CuO NWs is Cu_2_O.

Additionally, the relative proportions of Cu^1+^ and Cu^2+^ on the NW surface were analyzed by comparing the total integrated intensity of peaks. The relative atomic percentages are also presented in Figure 6. The Cu^1+^ atomic percentage initially demonstrates a significant increase, followed by a slight decrease with increasing ion fluence, whereas the Cu^2+^ atomic percent exhibits the opposite trend. This indicates that the degree of surface reduction does not continuously rise with increasing ion fluence. This phenomenon may be attributed to the rougher surface characteristics of irradiated NWs at higher ion fluences (>5 × 10^15^ ions/cm^2^), which significantly enhance their specific surface area; when exposed to air, these NWs demonstrate a greater affinity for oxygen compared to smoother NWs [44]. As a result, the reduced Cu^+^ species on the rougher surfaces are readily re-oxidized. Nevertheless, the exact mechanism underlying this phenomenon necessitates further investigation.

**Figure 7:**
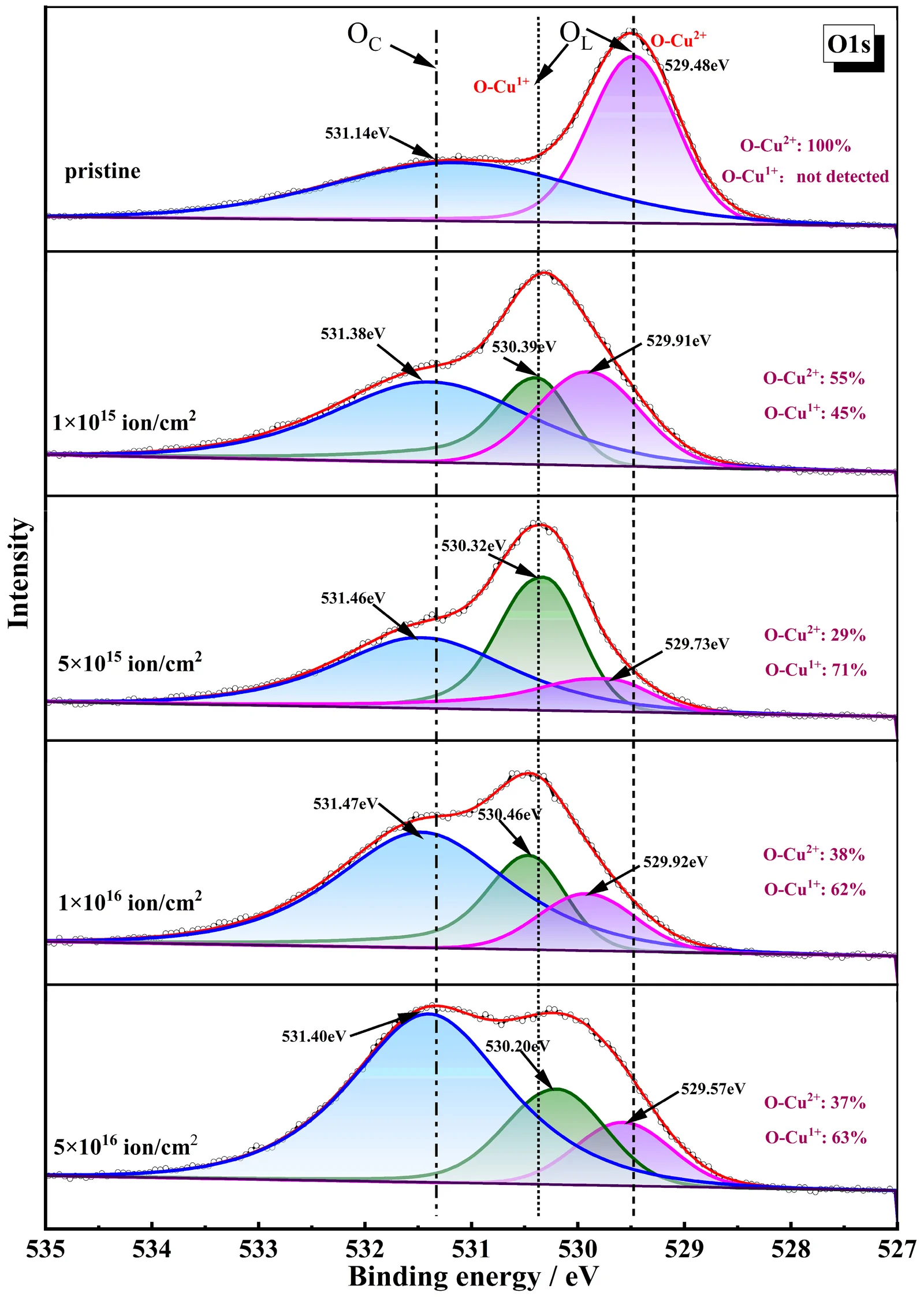
High-resolution XPS O1s spectra of CuO NWs before and after ion bombardment with different ion fluences.

The detailed O1s spectra for all NW samples are displayed in Figure 7. Before irradiation, the broad O1s peak is deconvoluted into two distinct peaks at 529.48 eV and 531.14 eV. The lower binding energy peak at 529.48 eV corresponds to the lattice oxygen (O_L_), originating from O atoms intrinsically bonded to Cu atoms within the CuO structure, while the higher binding energy peak at 531.14 eV is attributed to chemisorbed oxygen species, primarily arising from atmospheric hydroxides (O_C_) [45]. Following irradiation, the O_L_ and O_C_ peaks shift toward higher binding energies, likely due to the incorporation of Ti ions, which exert a stronger attraction toward electrons. The O_L_ peak intensity decreases while the O_C_ peak intensity increases, suggesting that collisions displace lattice O atoms, thereby creating oxygen vacancies and enhancing hydroxyl adsorption at these vacancy sites [42]. Furthermore, the O_L_ peak can be further deconvoluted into two sub-peaks corresponding to CuO (~529.91 eV) and Cu_2_O (~530.39 eV) [23], corroborating the partial reduction of the Cu^2+^ to Cu^+^ depicted in Figure 6.

From the above experimental observations, we have determined that 80 keV Ti ion irradiation significantly alters the morphology, microstructure, and surface chemical composition of CuO NWs. The morphological changes can be categorized into two aspects: bending and surface roughening. The significant microstructural modification is the amorphization of the NWs at higher fluences. The primary alteration in chemical composition involves the partial reduction of CuO to Cu_2_O on the NW surface. Actually, the above ion-beam-induced modifications have been observed in a variety of NWs. Table S1 summarizes the survey results, categorizing experiments by target material and highlighting the modifications in NWs under ion irradiation [23,24,35,39,42,46,47,48,49,50,51]. As shown in Table S1, a detailed comparison reveals that these phenomena strongly depend on the ion-beam parameters, including ion species, mass, energy, and fluence, as well as on the target material itself. In the case of CuO NWs [23,24], the surface morphologies obtained in this work exhibit marked differences from those induced by N, Fe, and Ar ion irradiation; the microscopic mechanisms responsible for CuO NW bending and roughening have not been adequately addressed in prior studies. To gain a deeper understanding of the effects of Ti ion beam on CuO NWs, it is necessary to conduct theoretical calculations to predict the trajectories, spatial distributions, and resulting defect distributions of energetic ions within the NWs.

### 3.4. Simulation Results

Based on the SEM results presented in Figure 2, the NW diameter ranges from approximately 30 to 90 nm. Accordingly, four distinct diameters (30, 50, 70 and 90 nm) were selected for simulations, with the ion energy (80 keV) and incidence angle (90°) held constant.

**Figure 8:**
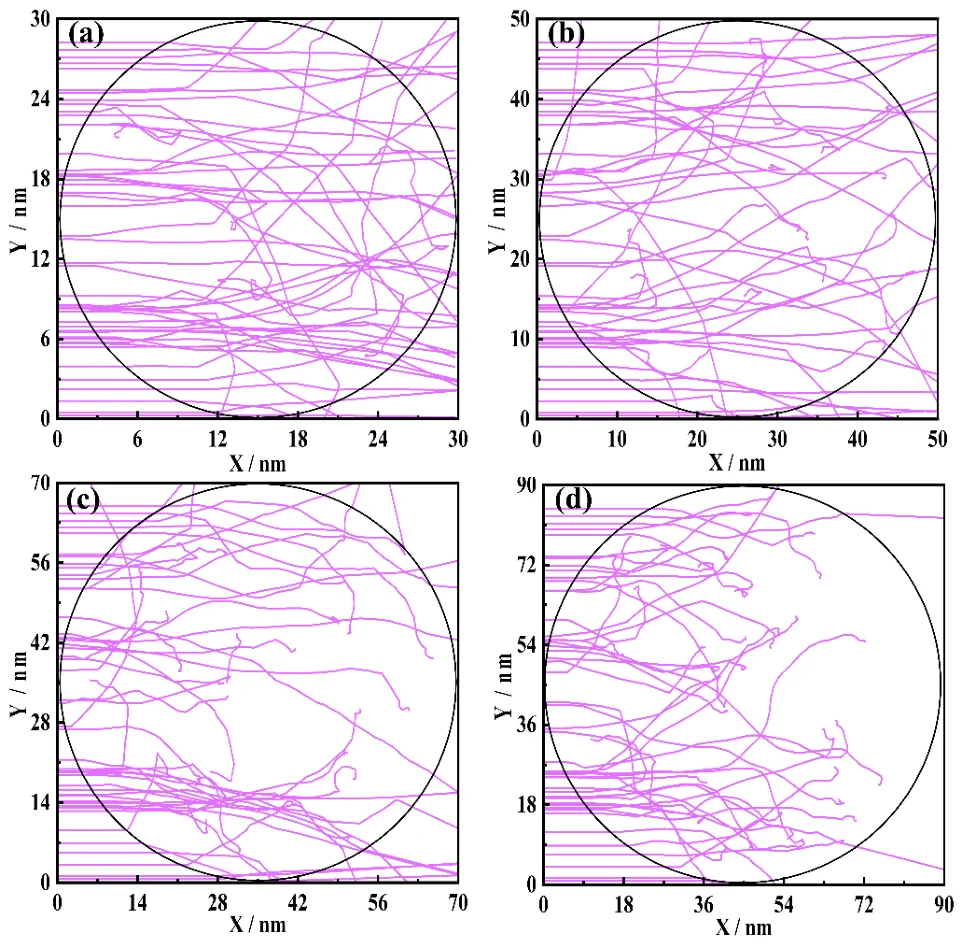
The trajectories of 80 keV Ti ions implanted into CuO NW with different diameters: (**a**) 30 nm; (**b**) 50 nm; (**c**) 70 nm; (**d**) 90 nm.

Figure 8a–d present the trajectories of 80 keV Ti ions within the cross-sections of NWs of varying diameters. The results reveal that as the diameter increases, the ion trajectories exhibit pronounced variations, which may be related to the ion range [35]. Notably, the average projected range of 80 keV Ti ions in bulk CuO, as calculated by SRIM, is approximately 45 nm. When the NW diameter is 30 nm (smaller than 45 nm), as shown in Figure 8a, a considerable fraction of Ti ions escapes laterally from or completely pass through the NW rather than being implanted. In contrast, for diameters larger than 37 nm [Figure 8b–d], most ions are eventually stopped within the NW.

**Figure 9:**
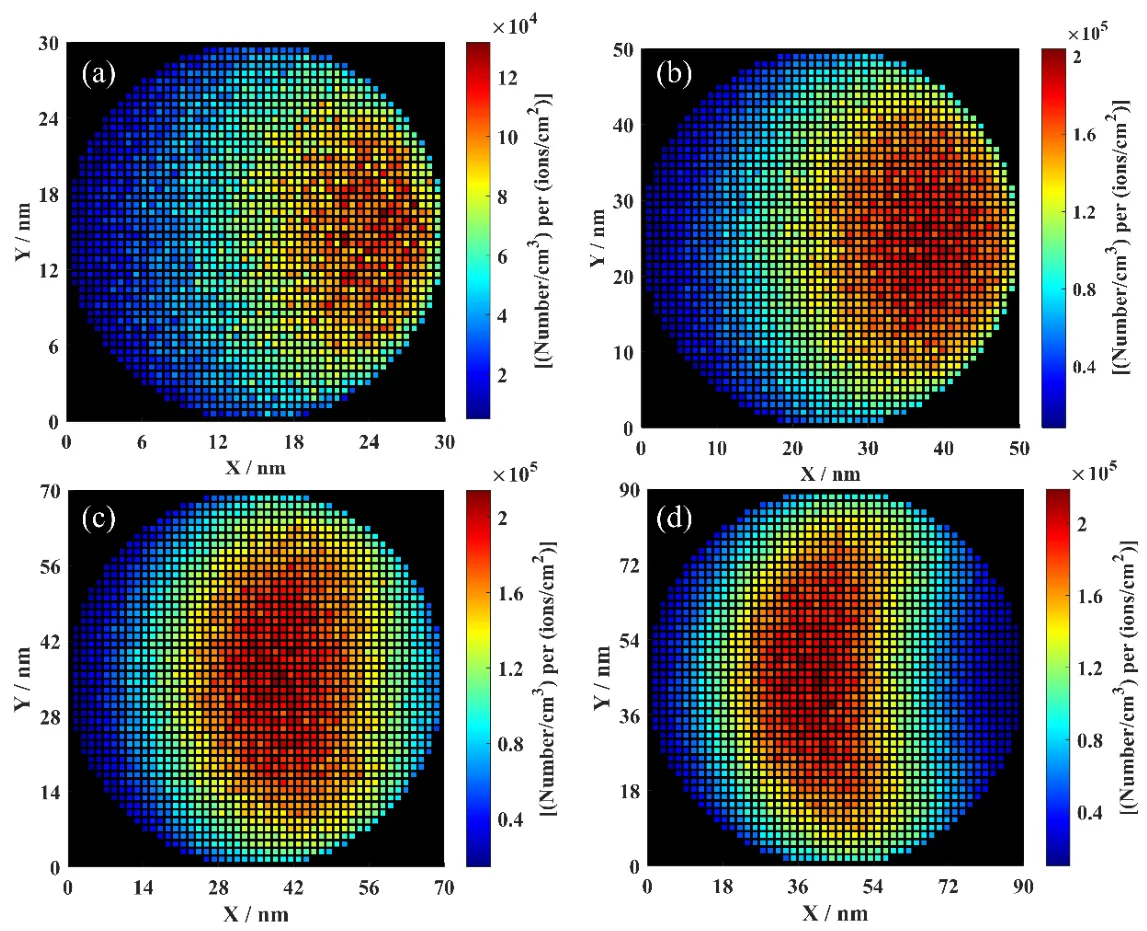
Distribution of range for 80 keV Ti ions implanted into CuO NW with different diameters: (**a**) 30 nm; (**b**) 50 nm; (**c**) 70 nm; (**d**) 90 nm. The unit of right color scale is [(Number/cm^3^) per ions/cm^2^].

Figure 9a–d show the cross-sectional distributions of Ti ions within the NWs following irradiation. The results indicate that the depth profile of Ti concentration varies systematically with NW diameter. For smaller diameters (30 nm and 50 nm) [Figure 9a,b], most ions are implanted into deeper regions of the NWs. In contrast, for larger diameters (70 nm and 90 nm) [Figure 9c,d], the predominant Ti concentration shifts toward shallower regions. Therefore, for a fixed ion energy, variation in NW diameter leads to distinctly different irradiation regimes: smaller diameters facilitate deeper ion penetration, whereas larger diameters result in shallower implantation. This indicates that the effective irradiation depth depends not only on the incident ion energy but also significantly on the NW diameter [48]. Furthermore, the volume concentration of Ti ions (normalized to unit fluence) in the 30 nm NW is approximately one order of magnitude lower than that in NWs of larger diameters [Figure 9a–d]. This significant reduction is due to the substantial escape of incident ions from the NW when its diameter falls below the projected range (i.e., <45 nm), as clearly demonstrated in Figure 8a.

**Figure 10:**
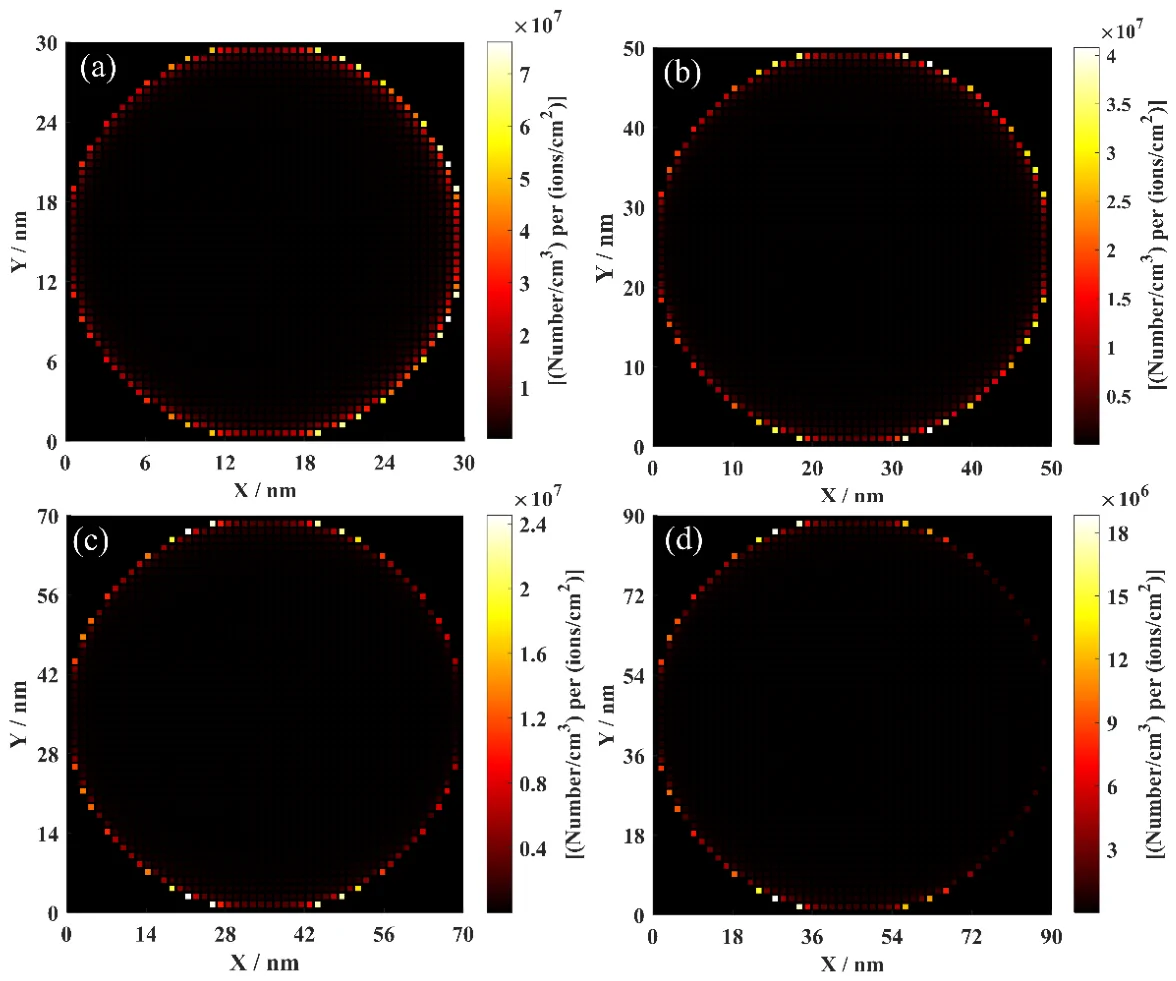
Distribution of surface sputtered atoms produced by 80 keV Ti ions implanted into CuO NW with different diameters: (**a**) 30 nm; (**b**) 50 nm; (**c**) 70 nm; (**d**) 90 nm. The unit of right color scale is [(Number/cm^3^) per ions/cm^2^].

Figure 10a–d demonstrate the distributions of sputtered atoms within the irradiated NW samples. Sputtering refers to the ejection of surface atoms primarily through elastic collision processes [28]. As shown in Figure 10a,b, when the NW diameter is 30 or 50 nm, a large number of atoms are sputtered from the entire NW surface. However, as the NW diameter increases to 70 or 90 nm [Figure 10c,d], sputtering becomes confined to only part of the surface, with fewer atoms ejected, as indicated by the color scale on the right. This diameter-dependent behavior arises because, for smaller diameter NWs (30 and 50 nm), whose dimensions are comparable to the projected range of ~45 nm; although most of the ion energy is deposited within the NW, a considerable portion of the kinetic energy from collision cascades still reaches the surface, thereby inducing sputtering across the entire NW surface [35]. In contrast, for larger diameters (70 and 90 nm) [Figure 10c,d], ions incident on the central region lose all their energy and come to rest several nanometers away from the surface. Consequently, they cannot transfer sufficient energy to surface atoms and thus do not contribute to sputtering in those regions [31].

We further calculated the sputtering yield of the NW samples. The total sputtering yield (*Y_Tol_*) of a compound is defined as the number of sputtered atoms per incident ion (atoms/ion) [31], encompassing all constituent species, including Cu and O atoms. The calculated values of *Y_Tol_* are summarized in Table 1. For diameters ranging from 30 to 90 nm in diameters, *Y_Tol_* exhibits a decreasing trend with increasing diameters, indicating that the *Y_Tol_* strongly depends on the NW diameter. In addition, the number of sputtered O atoms consistently exceeds that of Cu atoms across all diameters. This result is in agreement with the theory of preferential sputtering in oxides, which attributes the preferential ejection of oxygen primarily to the significant mass difference between the metal and oxygen atoms [23].

**Figure 11:**
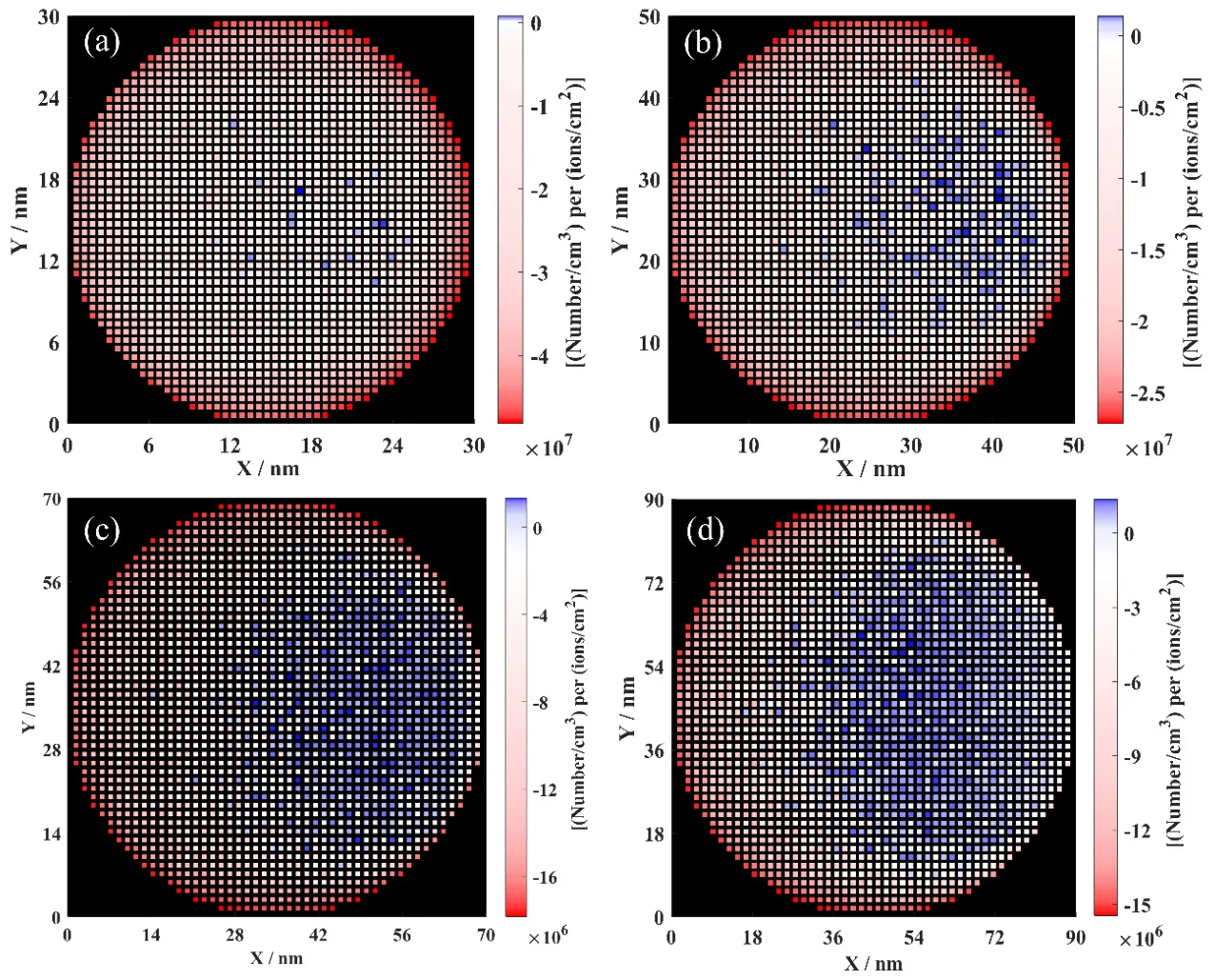
Distribution of remaining defects produced by 80 keV Ti ions implanted into CuO NW with different diameters: (**a**) 30 nm; (**b**) 50 nm; (**c**) 70 nm; (**d**) 90 nm. The unit of right color scale is [(Number/cm^3^) per ions/cm^2^]. Remaining defects are calculated as interstitials minus vacancies. Blue: excess interstitials, red: excess vacancies.

Figure 11a–d present the distributions of remaining defects within the irradiated NW samples (Red shows excess vacancies, blue shows excess interstitials). In the Iradina simulation, defects are assumed to annihilate only within each simulation cell, with no annihilation across cell boundaries [35]. Consequently, the number of remaining defects in each cell is approximately given by the difference between the number of interstitials and vacancies. This approximation is considered sufficient for qualitative analysis. As the diameter increases, the depth profile of remaining defects experiences significant alterations. For smaller diameters (30 and 50 nm) [Figure 11a,b], defects are generated across the entire cross-section of the NW; however, excess interstitials accumulate at greater depths, while excess vacancies concentrate on the ion-beam-facing side. For larger diameters (70 and 90 nm) [Figure 11c,d], the defect distribution shifts markedly toward the shallower region, i.e., the side facing the ion beam. Notably, the spatial distribution of excess interstitials consistently remains slightly deeper than that of excess vacancies.

This phenomenon can be attributed to the directionality of atomic collision cascades [36], which is governed by the trajectories of the incident ions. In each knock-on event within a collision cascade, a vacancy and an interstitial are simultaneously created; however, the vacancy stays at the collision site, whereas the interstitial is displaced forward due to the momentum transfer from the incoming ion [35]. This fact leads to a spatial separation between the two types of defects. Although vacancies and interstitials can effectively recombine at room temperature, the excess vacancies consistently remain in the shallower region of the cascade, while the excess interstitials are retained at greater depths.

In addition, the concentration of excess vacancies significantly exceeds that of excess interstitials throughout the NWs [Figure 11a–d]. This disparity arises because the formation energy of a vacancy is considerably lower than that of an interstitial [52]. A closer inspection reveals that an unrealistically high concentration of excess vacancies appears near the NW surface. This artifact is attributed to the static nature of the Iradina code, in which the surface remains fixed during the simulation [35]. In reality, when sputtering is taken into account, this vacancy-rich surface layer would be directly removed during irradiation [35].

### 3.5. Discussions

By combining theoretical simulations with experimental observations, we focus on several key issues concerning the Ti-beam-induced modifications of CuO NWs. Firstly, the pronounced bending observed under ion irradiation is of particular interest, as it offers promising routes for controlled NW engineering. As evident from the SEM images in Figure 2, the NWs undergo noticeable bending following Ti ion irradiation: the degree of bending is strongly dependent on ion fluence; at sufficiently high fluence, the bending at the tip reverses direction, resulting in S-shaped NWs. This bending phenomenon has been widely observed across various NW systems. And several possible mechanisms have been proposed to account for this effect, including ion-implantation-induced expansion, density changes, complete amorphization, spatial separation of vacancies and interstitials, sputtering, and viscoelastic flow [36]. However, a definitive consensus has yet to be established.

**Figure 12:**
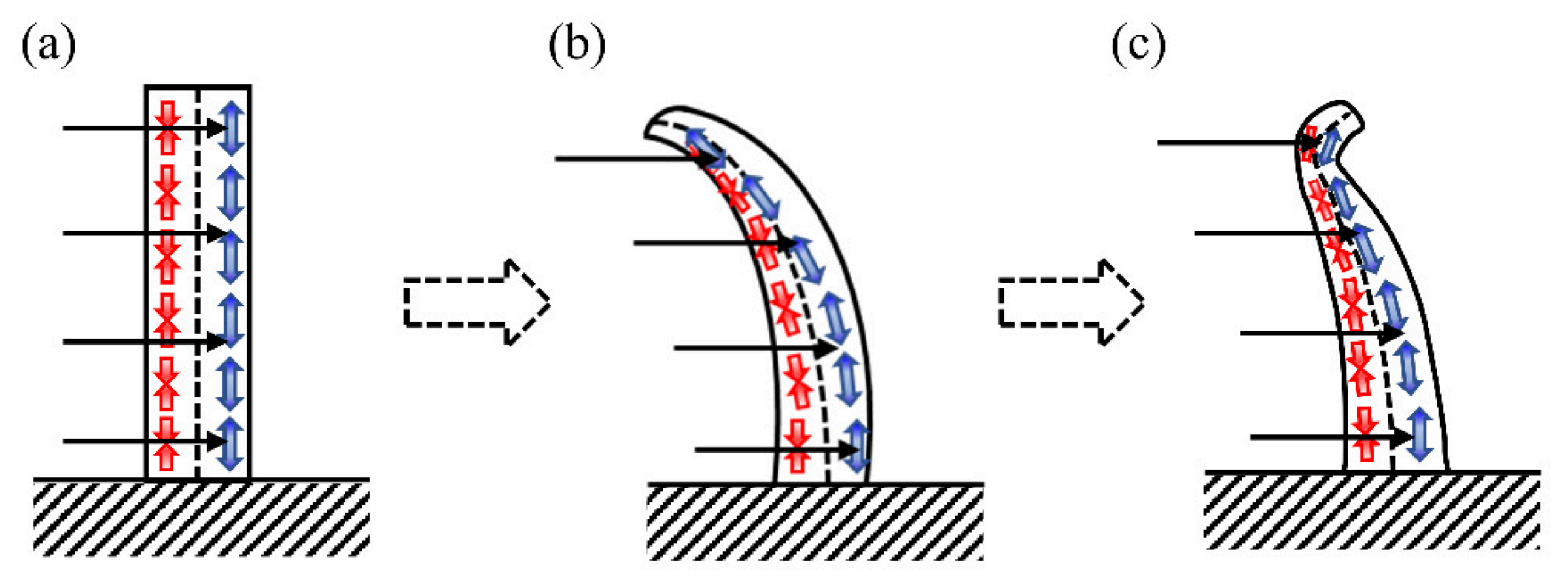
A schematic illustration of the bending process of CuO NW under 80 keV Ti ion beam irradiation: (**a**) initially straight NW is irradiated homogeneously. Contraction on left side (red arrows), expansion on the right side (blue arrows); (**b**) the NW bends towards the ion beam due to the resultant bending moment; (**c**) with bending, the angle between the ion beam and the NW changes, the expansion/contraction regions shift, and the bending at the tip reverses.

In view of the used ion energy (80 keV) and the fact that the present experiment was conducted on NW arrays (as opposed to a single NW), the spatial separation mechanism of vacancies and interstitials offers a plausible interpretation of our experimental findings. A schematic diagram of this mechanism is presented in Figure 12. According to the simulation results of the defects in Figure 11, excess vacancies are predominantly concentrated on the ion-beam-facing side, inducing volume contraction (red arrows) [Figure 12a]; conversely, excess interstitials accumulate on the backside, leading to volume expansion (blue arrows) [Figure 12a]. The resultant bending moment causes the NW to bend towards the ion beam [Figure 12b]. Meanwhile, the NWs become slightly thinner due to sputtering effect. As bending progresses, the angle between the NW tip and the ion beam decreases significantly, thereby reducing the ion projected range and shifting the defect distribution near the tip [Figure 12b] [53]. This reverses the bending moment, inducing opposite bending at the tip and ultimately giving rise to S-shaped NWs [Figure 12c]. In contrast, the NW base remains firmly attached to the substrate, with the angle between the ion beam and the NW base remaining nearly constant. Therefore, by adjusting the ion fluence, ion beam irradiation enables controlled bending and subsequent re-alignment of the NW axis during post-growth processing.

**Figure 13:**
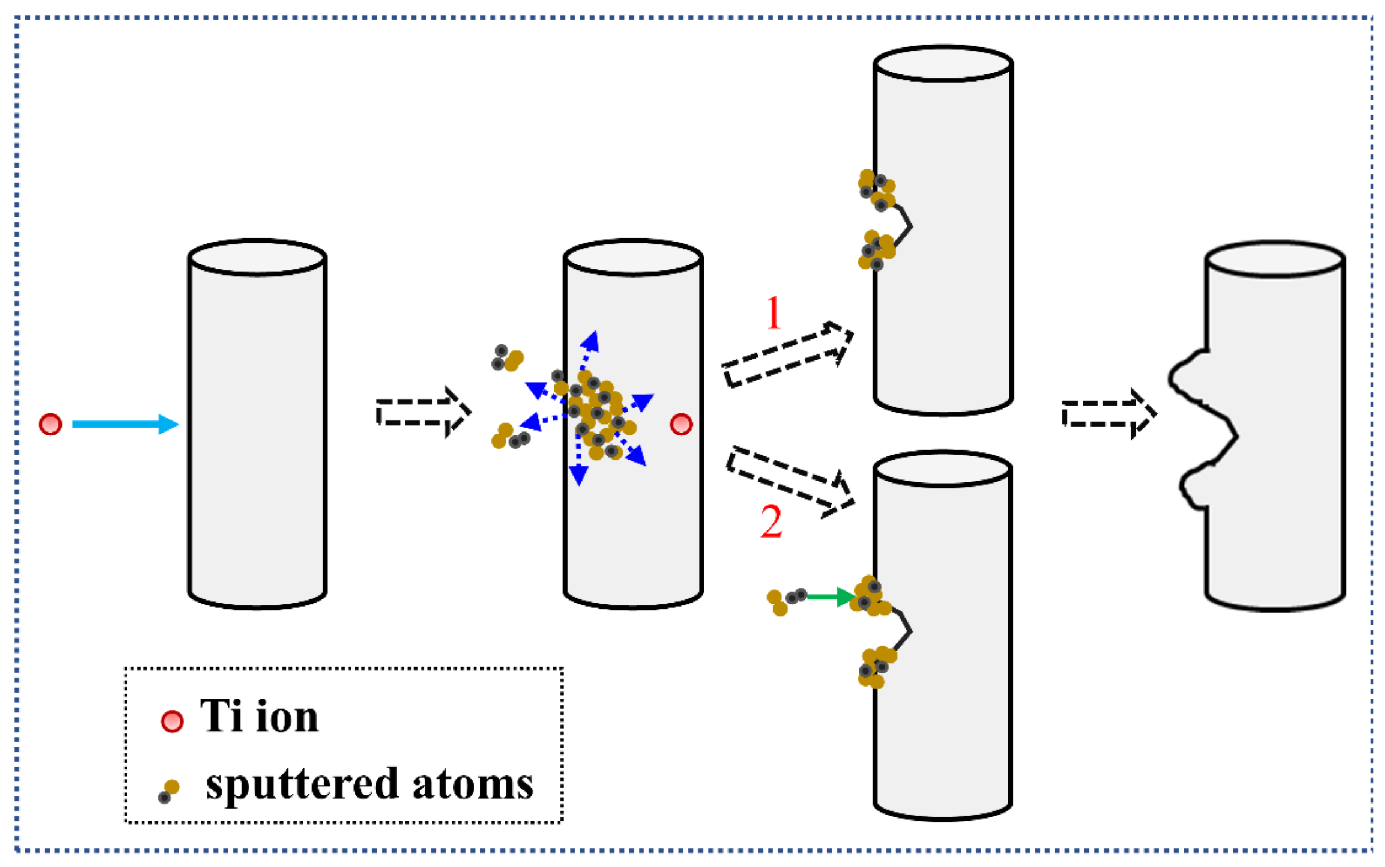
A sketch map of the formation process of the craters and protrusions on the surface of irradiated CuO NW. Sputtered atoms form protrusions near a crater by either (1) direct adhesion when their kinetic energy is below the surface adsorption energy, or (2) escape and subsequent redeposition when their kinetic energy is sufficiently high.

Secondly, ion-beam-induced roughening modifies the surface morphology of the NWs. It is a common phenomenon during ion irradiation of nanomaterials and is often broadly attributed to sputtering effect [27,28,43,45]. In fact, sputtering will lead to surface erosion; however, it does not always constitute an undesired damaging effect [53]. Based on the experimental observations [Figure 3 and Figure 4], it is evident that 80 keV Ti beam irradiation produces jagged NW contours through the formation of craters and protrusions on their surfaces, thereby introducing surface roughness. Indeed, the emergence of such new morphologies suggests frequent mass transport [49]. A sketch map of the formation process is illustrated in Figure 13.

Owing to sputtering, when energetic ions collide with the NWs, surface-near target atoms may acquire sufficient energy to be ejected from the NWs, resulting in the formation of surface depressions. Such depressions can locally increase the sputtering yield, creating a positive feedback loop for further depression development [53]. The simulation results of the sputtering in Table 1 indicate that the number of sputtered atoms increases with rising fluence; accordingly, local depressions evolve into craters as fluence increases. Moreover, two possible explanations are proposed for the formation of protrusions [Figure 13]: (1) if the kinetic energy of the sputtered atoms is lower than the adsorption energy of the NW surface, these atoms may directly adhere to the surface, forming protrusions near the crater; (2) when their kinetic energy is sufficiently high, the sputtered atoms escape from the NW and may subsequently redeposit on the surface as protrusions adjacent to the crater [50]. In addition, due to the statistical nature of sputtering—characterized by a broad distribution of the number of sputtered atoms per incident ion, the roughening of the NW surface is inherently non-uniform [53].

**Figure 14:**
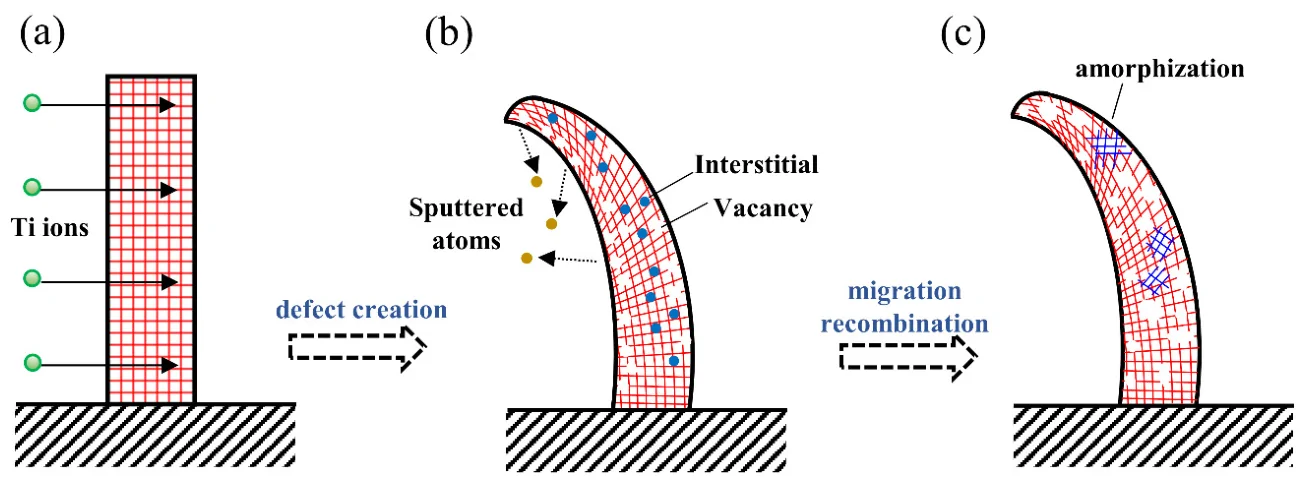
A schematic illustration of the amorphization process of CuO NW under 80 keV Ti ion beam irradiation: (**a**) energetic-ions impact on the NW; (**b**) generation of numerous vacancies and interstitials by ion bombardment; (**c**) localized amorphization of the NW induced by defect migration and recombination.

Thirdly, the TEM observations in Figure 4 reveal the formation of an amorphous shell surrounding a crystalline core following irradiation; as fluence increases, the thickness of the shell layer exhibits a certain rising trend. We conduct a detailed analysis of the amorphization behavior of NWs under ion irradiation. This behavior is primarily governed by the generation, migration, and recombination of defects, as schematically depicted in Figure 14. As shown in Figure 14a,b, energetic ion bombardment displaces the target atoms in the NW, producing numerous vacancies and interstitials. Figure 14c shows that the NW surface acts as an effective trap for migrating defects; the accumulation of defect complexes at the surface raises the free energy, thereby providing nucleation sites for amorphization, which then collapses into an energetically favorable amorphous state [53]. The defect simulation results (Figure 10) corroborate this conclusion: within the investigated diameter range, the defect concentration near the surface is considerably higher than in the core. Additionally, dynamic annealing occurs during ion irradiation and promotes defect recombination [51]. However, once the irradiation fluence exceeds a critical threshold, the defect generation rate begins to substantially overtakes that of recombination. Consequently, this net increase in defect concentration promotes the growth of the amorphous layer (in thickness or volume fraction) with increasing fluence.

Finally, another notable finding concerns the surface chemical changes of the NWs following Ti ion irradiation. The XPS results in Figure 6 and Figure 7 reveal a decreasing trend in lattice oxygen content and a partial reduction from CuO to Cu_2_O. Concurrently, the sputtering simulation results (Table 1) demonstrate that more O atoms than that of Cu atoms are sputtered from the NW surface during irradiation. This consistency supports the theory of preferential sputtering of metal oxides, which attributes the preferential loss of oxygen to its lower atomic mass [23,43]. Furthermore, the observed reduction can also be accounted for by the thermal spike model [53]. The elevated temperature generated within the thermal spike may induce thermodynamic instability of CuO [54]^,^ thereby promoting its decomposition into Cu_2_O [43]. Overall, these combined experimental and simulation results provide a more comprehensive understanding of the alterations in the irradiated NWs.

### 3.6. Wettability Measurements

**Figure 15:**
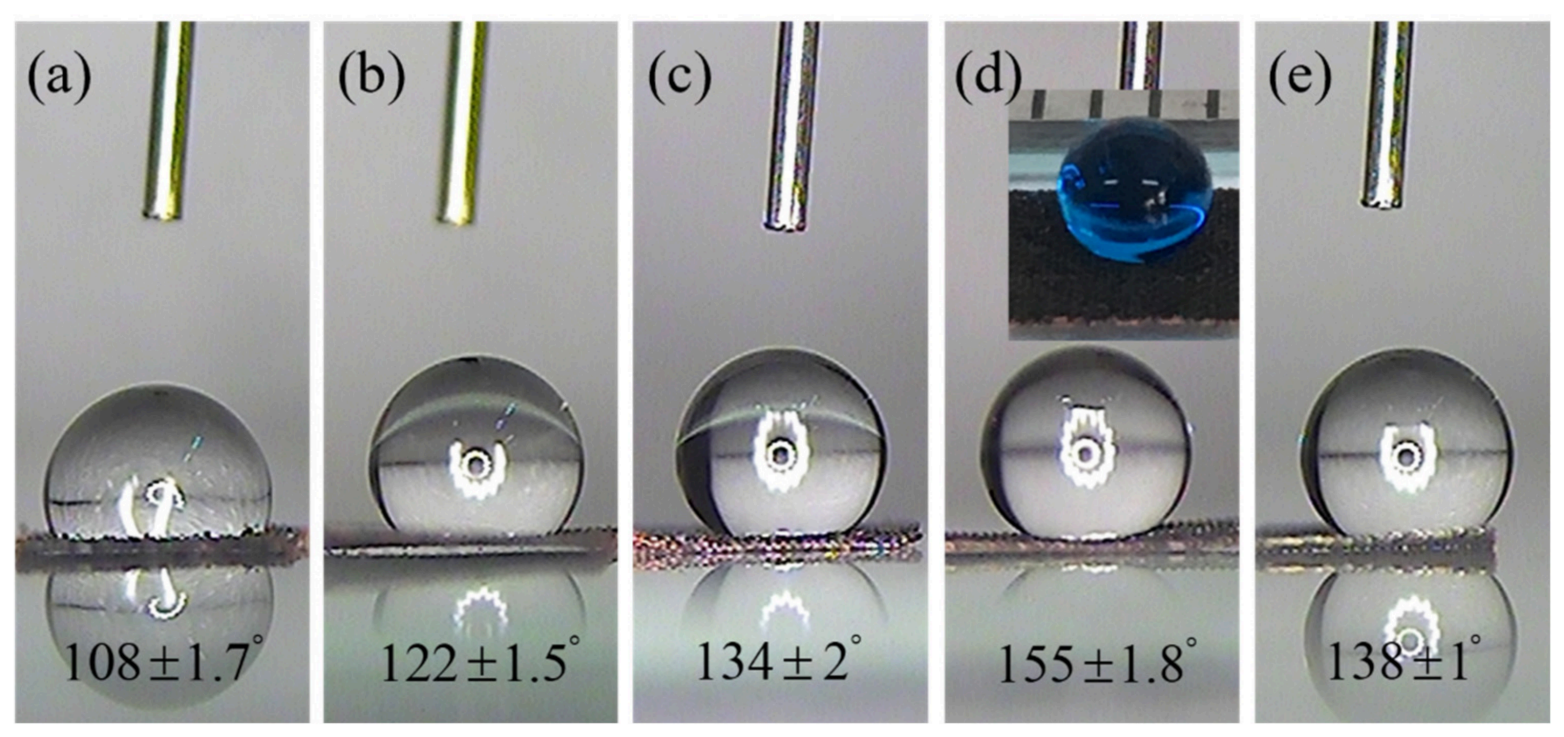
Static water contact angle (SWCA) of a water droplet on the surfaces of 80 keV Ti ion irradiated CuO NWs with different fluences: (**a**) 0; (**b**) 1 × 10^15^; (**c**) 5 × 10^15^; (**d**) 1 × 10^16^; (**e**) 5 × 10^16^ ions/cm^2^. Inserted image shows the photograph of a water droplet (dyed with methylene blue) on the irradiated NW surface at a fluence of 1 × 10^16^ ions/cm^2^.

The surface wetting behaviors of CuO NWs before and after Ti ion irradiation were investigated through contact angle measurements, with the results presented in Figure 15.

As shown in Figure 15a, the pristine NW surface exhibits weak hydrophobicity, with a static water contact angle (SWCA) of 108 ± 1.7°. It is noteworthy that bulk CuO is typically hydrophilic [55]; however, the CuO NWs in our study display hydrophobicity. With increasing ion fluence, as illustrated in Figure 15b–e, the SWCA initially increases and then decreases slightly. When the fluence reaches 1 × 10^16^ ions/cm^2^ [Figure 15d], the SWCA attains a maximum value of 155 ± 1.8°. Under this condition, a water droplet dyed with methylene blue maintains a spherical shape on the surface (see the inserted image). Moreover, when this NW sample is placed on an inclined plane with a small angle, the water droplet rolls off the surface easily and rapidly (see Supporting Information Video S1), consistent with the definition of super-hydrophobicity [56]. These findings indicate that ion irradiation enhances the hydrophobic properties of the NW surface, and that the optimal ion fluence can be tuned to achieve a superhydrophobic NW surface.

**Figure 16:**
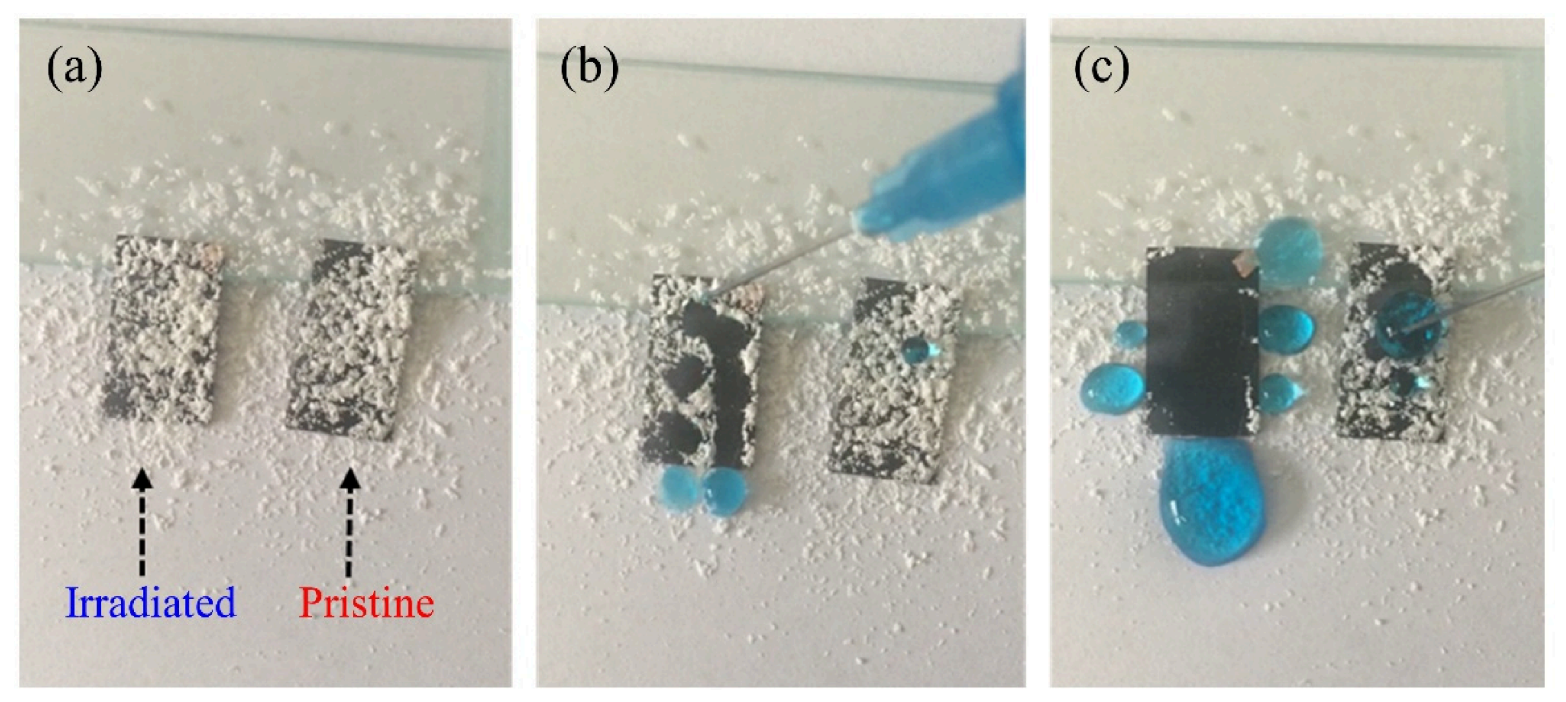
Digital photographs of the self-cleaning process for the irradiated CuO NWs (1 × 10^16^ ions/cm^2^) with superhydrophobic surfaces: (**a**) before; (**b**) during; (**c**) after cleaning with water.

We further investigated the self-cleaning property of the irradiated NWs with superhydrophobic surfaces (see Figure 16 and Video S2). As shown in Figure 16a, the NW samples were leaned against a glass slide (tilting angle ~2°), with a sparse layer of white chalk debris uniformly coated on their surface. In Figure 16b,c, when water droplets were slowly placed onto the irradiated NW sample, they rolled away instantly together with the debris, leaving behind a clean trail. In contrast, water droplets remained stationary and adhered firmly to the surface of the pristine NW sample. These results demonstrate that the irradiated NWs with superhydrophobic surfaces possess effective self-cleaning properties and resistance to contamination, highlighting their advantages for practical applications [57].

A possible mechanism is proposed to elucidate the wettability change of the NW surfaces under Ti ion beam irradiation. Specifically, the wettability of Ti-irradiated CuO NWs is governed by two effects: a morphological effect (related to surface roughness) and a chemical effect (related to surface composition). Their combined effect determines the wetting behavior across different fluences, as summarized in Table 2.

**Figure 17:**
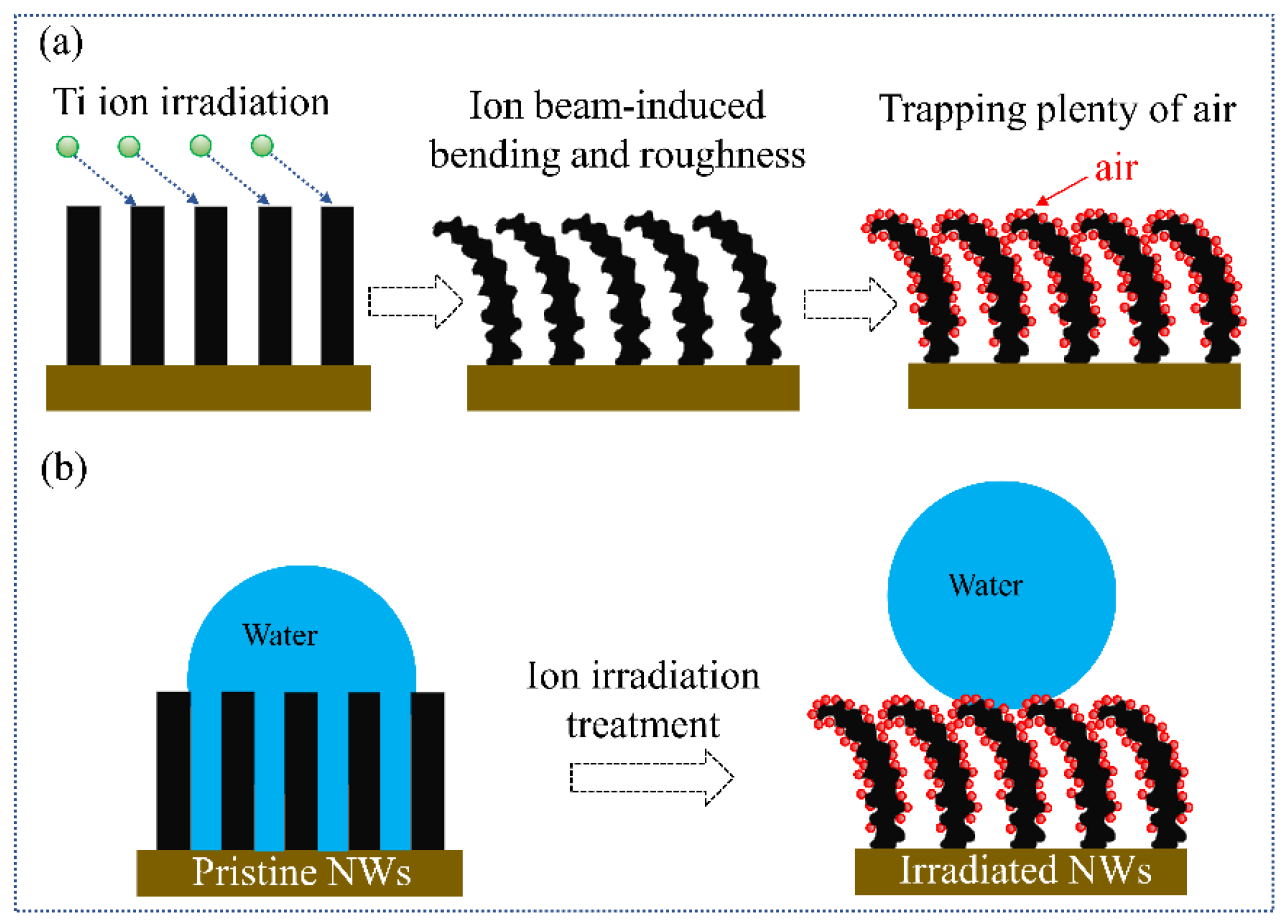
Schematic illustration of the mechanism of wettability evolution for CuO NW surfaces before and after Ti ion irradiation: (**a**) morphological and structural evolution process of NW; (**b**) the wetting state evolution of water droplet on the NW surface.

**Figure 18:**
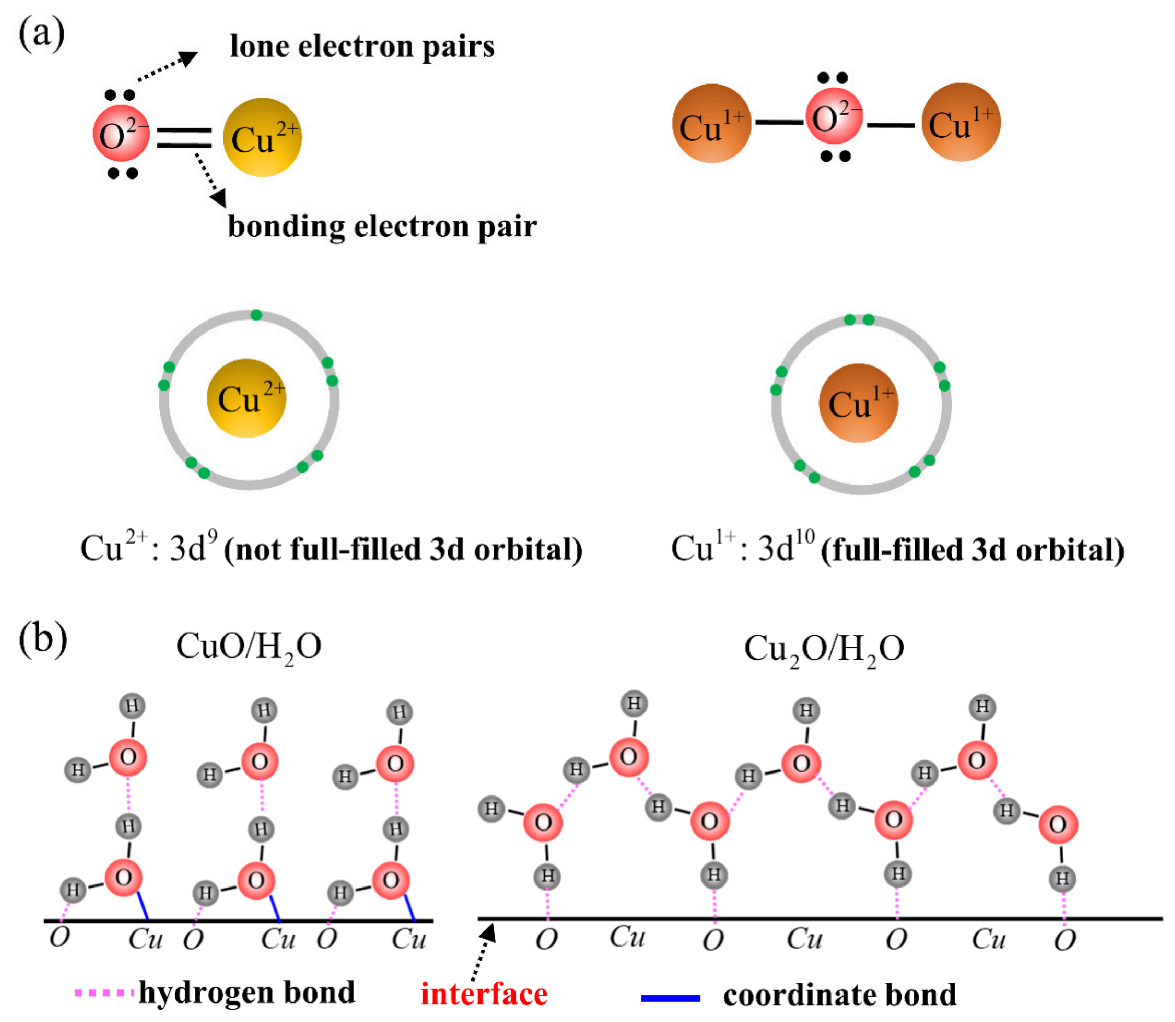
Characteristics determining the hydrophilicity or hydrophobicity of CuO NW surfaces before and after Ti ion irradiation: (**a**) schematic of the bonding of an oxygen anion (O^2−^) to a copper (Cu^2+^) cation in CuO and a copper (Cu^1+^) cation in Cu_2_O, respectively; (**b**) schematic of the water affinity with CuO surface and Cu_2_O surface, respectively.

At low fluences (≤1 × 10^16^ ions/cm^2^), both effects act in the same direction to enhance hydrophobicity. As shown in Figure 17, Ti ion irradiation induces bending of the NWs, causing them to form arch-like structures, while protrusions and craters increase the surface roughness [Figure 17a]. These unique micro/nanostructures trap a greater amount of air beneath the water droplet, reducing the solid–liquid contact area and increasing the SWCA [Figure 17b], in accordance with the Cassie–Baxter model [58]. In addition, irradiation also reduces a portion of CuO to Cu_2_O. As illustrated in Figure 18, a Cu^2+^ in CuO is electron-deficient (3d^9^) and acts as a Lewis acid (electron-pair acceptor), while a O^2−^ in CuO has two nonbonding lone electron pairs and acts as a Lewis base (electron-pair donor) [Figure 18a]. Together, they exhibit a high chemical affinity for water molecules by forming coordinate bonds (blue line) and hydrogen bonds (purple line), rendering CuO hydrophilic [left of Figure 18b]. In contrast, a Cu^1+^ in Cu_2_O has a fully filled 3d^10^ orbital and thus cannot accept electrons to form coordinate bonds [right of Figure 18b]. Consequently, with only hydrogen bonds available, the Cu_2_O surface has a low affinity for water molecules and is therefore hydrophobic [59,60]. The partial conversion of CuO to Cu_2_O therefore lowers the surface energy and enhances hydrophobicity. The simultaneous increase in roughness (morphological effect) and decrease in surface energy (chemical effect) leads to the maximum SWCA of 155 ± 1.8° at fluence of 1 × 10^16^ ions/cm^2^.

**Figure 19:**
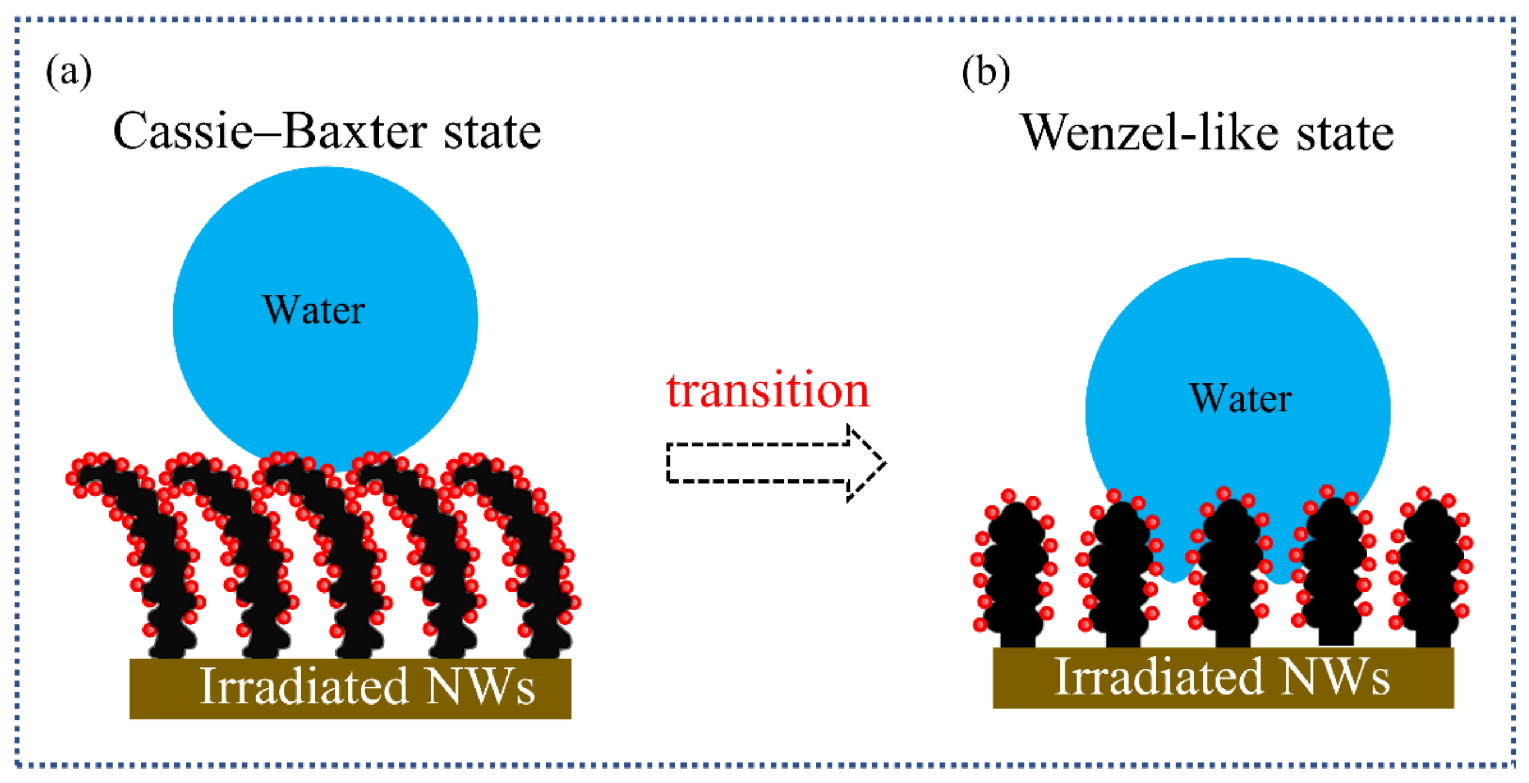
Schematic illustration of the transition from Cassie–Baxter state to a Wenzel-like state for a water droplet on the irradiated CuO NW surface: (**a**) low-fluence irradiated NWs; (**b**) high-fluence irradiated NWs.

At high fluences (>1 × 10^16^ ions/cm^2^), however, both effects act in the opposite direction, leading to a slight decrease in SWCA [Figure 15e]. Morphologically, the protrusions and craters progressively grow and coalesce, the NW tips begin to fracture, and the arch-like structures are destroyed [Figure 2e and Figure 3e]. As a result, the density of air-trapping sites decreases and the spacing between adjacent NWs increases, allowing water to penetrate the surface asperities. This may favor a transition from the Cassie–Baxter state toward a Wenzel-like state (an intermediate state) [61], as shown in Figure 19, which in turn decreases the SWCA. Chemically, XPS analysis (Figure 6) shows that the Cu^1+^ atomic percentage first increases and then slightly decreases with ion fluence, indicating partial re-oxidation of Cu_2_O back to CuO. Because CuO is more hydrophilic than Cu_2_O, this re-oxidation further contributes to the SWCA decrease. The decline in SWCA at high fluences thus stems from two concurrent processes: morphological coarsening, which eliminates air-trapping structures, and chemical re-oxidation, which raises the surface energy. In other words, both the morphological and chemical effects reverse their contribution at high fluences, acting together to weaken hydrophobicity.

This mechanism may help explain the observed fluence-dependent wetting behavior and provide a basis for optimizing irradiation conditions to achieve stable superhydrophobicity.

## 4. Conclusions

In this work, CuO NWs were irradiated with an 80 keV Ti ion beam at four fluences (1 × 10^15^, 5 × 10^15^, 1 × 10^16^, and 5 × 10^16^ ions/cm^2^), and the resulting surface modifications were investigated through combined experimental observations and Iradina simulations. The combination of the experimental and simulated results establishes a clear correlation between the defect structure and the properties of CuO NWs, and provides atomic-level insights into the mechanisms of ion-beam-induced modifications. The main findings are summarized as follows:

(i) Bending was observed experimentally after irradiation, and the spatial separation of vacancy and interstitial distributions, revealed by defect simulations, is identified as the dominant mechanism.
(ii) Surface roughening arose from the formation of craters and protrusions. Sputtering simulations showed that the number of sputtered atoms increases with ion fluence, supporting surface sputtering as the underlying cause.
(iii) The partial reduction of CuO to Cu_2_O was detected by XPS. Calculations of the sputtering yield indicated preferential loss of oxygen, while the thermal spike model accounted for the thermal decomposition; together, these two factors explained the observed reduction.
(iv) Amorphization was observed as a surface amorphous layer. Defect simulations showed that defects accumulate preferentially near the surface, and dynamic annealing governed their migration and recombination, driving the amorphization process.
(v) The fluence-dependent wetting behavior was observed experimentally. Its mechanism was attributed to the combined action of a morphological effect (related to surface roughness) and a chemical effect (related to surface composition).

## Figures and Tables

**Table 1:** The number of sputtered Cu atoms and O atoms, the total sputtering yield (*Y_Tol_*) of CuO NWs irradiated by 80 keV Ti ions with different diameters.

Species of Sputtered Atoms	Diameter of CuO NW/(nm)			
	30	50	70	90
O	8,110,000	7,643,362	6,124,165	5,112,426
Cu	7,725,974	7,189,018	5,732,347	4,785,747
*Y_Tol_* (atoms/ion)	15.84	14.83	11.85	9.89

**Table 2:** Summary of morphological effect, chemical effect, combined effects, and wetting state of CuO NWs under low- and high-fluence irradiation.

	Low-Fluence Irradiation (≤1 × 10^16^ ions/cm^2^)	High-Fluence Irradiation (>1 × 10^16^ ions/cm^2^)
Morphological Effect	increase in roughness; arch-like structures	decrease in roughness; destroyed arch-like structures
Chemical Effect	CuO→Cu_2_O, reduction; reduced surface energy	Cu_2_O→CuO, re-oxided; increased surface energy
Combined Effect	increased SWCA; enhanced hydrophobicity	decreased SWCA; reduced hydrophobicity
Wetting State	Cassie–Baxter	Wenzel-like

Note: “Wenzel-like” refers to a state in which the air layer is partially disrupted but may not be completely eliminated, and the liquid droplet partially penetrates the rough structures without fully wetting them.

## Data Availability

The original contributions presented in this study are included in the article/Supplementary Material. Further inquiries can be directed to the corresponding author.

## Supplementary Materials

The following supporting information can be downloaded at: https://www.mdpi.com/article/10.3390/ma19204289/s1, Table S1: Summary of the experiments on ion irradiation-induced modifications of various NWs reported in the literature, along with the results presented herein; Figure S1: High-resolution XPS Ti 2p spectra of CuO NWs after Ti ion bombardment with different ion fluences; Video S1: Superhydrophobicity test on ion-irradiated CuO NWs; Video S2: Self-cleaning process on ion-irradiated superhydrophobic CuO NWs.
