# Supplementary material for: Low-Energy Ti Ion Irradiation-Induced Modifications in CuO Nanowires: Experimental Measurements and Computational Simulations with a 3D Monte Carlo Code
Source: Materials (Basel). 2026 Oct 11;19(20):4289. doi: 10.3390/ma19204289 (PMC13707353; doi:10.3390/ma19204289)
Supplement: Supplementary file 1 [file materials-19-04289-s001.zip › Table S1 and Figure S1.pdf]

Table S1. Summary of the experiments on ion irradiation-induced modifications of various NWs reported in the literature, along with the results presented herein.

Ref.	Nanowire		Irradiation conditions			Before irradiation			After irradiation		
	Material	Ø/nm	Ion	Energy /keV	Fluence /ion.cm^{-2}	Morphology	Crystal structure	Surface composition	Morphology	Crystal structure	Surface composition
[23]	CuO	10~60	Ar	0.115 ~0.415	1.68×10^{17}	rough, straight, flat tip;	monoclinic crystalline;	CuO^-	smooth, bending, thinner, sharper tip;	partial amorphization	Cu^+/ Cu^{2+}
[24]	CuO	20~60	N	60; 100	1×10^{13}~2.5×10^{16}	smooth, straight;	monoclinic crystalline;	CuO	smooth, bending;	partial amorphization	Cu^+/ Cu^{2+}/N^{4+}
[24]	CuO	20~60	Fe	60; 100	1×10^{13}~2.5×10^{16}	smooth, straight;	monoclinic crystalline;	CuO	rougher, thicker, bending;	partial amorphization	Cu^+/ Cu^{2+}/Fe^{5+}
**This work**	CuO	30~90	Ti	80	1×10^{15}~5×10^{16}	smooth, straight, flat tip;	monoclinic crystalline;	CuO	rougher, bending, thinner, sharper tip;	partial amorphization	Cu^+/ Cu^{2+}/Ti^{4+}
[35]	GaAs	60	Mn	60	9.5×10^{15}	rough, straight;	single crystalline;	GaAs	smooth, bending;	complete amorphization	—
[39]	Bi	80,130	Au	400; 1000	1×10^{14} ~1×10^{15}	smooth, straight;	polycrystalline;	Bi	severe rougher, narrowing	amorphization, fragmentation;	bismuth oxide

									and necking;		
[42]	TiO_2	80-100	Mg, Co, Nb, W	8	1×10^{17}	smooth, straight, steeple-like tip;	Rutile crystalline;	TiO_2	No apparent changes	decreasing crystallinity;	Mg^{2+}/Co^{2+}/ Nb^{5+}/W^{6+}/ Ti^{3+}/ Ti^{4+}
[46]	TiO_2	20~40	N	45; 65; 85	1×10^{17}	smooth, straight;	mixed crystalline of anatase and rutile;	TiO_2	coarsening, straight;	decreasing crystallinity;	Ti^{3+}/ Ti^{4+}/N^{3-}
[47]	TiO_2	150	N	40	1×10^{15} ~1×10^{16}	smooth, straight;	Rutile crystalline;	TiO_2	smooth, straight;	decreasing crystallinity;	—
[48]	ZnO	50~100	Ti	30	2×10^{18}	smooth, straight;	single crystalline;	ZnO	smooth, straight;	decreasing crystallinity;	—
[49]	Au	20~85	Xe	1400	1×10^{14}	smooth, straight;	single crystalline;	Au	rougher, straight;	polycrystalline;	—
[50]	ZnO	60,90	Ar	20; 100	5×10^{14} ~1.2×10^{16}	smooth, straight;	single crystalline;	ZnO	smooth, bending;	decreasing crystallinity;	—
[51]	GaN	25~100	Ga	50	1×10^{14} ~2×10^{16}	smooth, straight;	single crystalline;	GaN	rougher	large-scale amorphization;	—

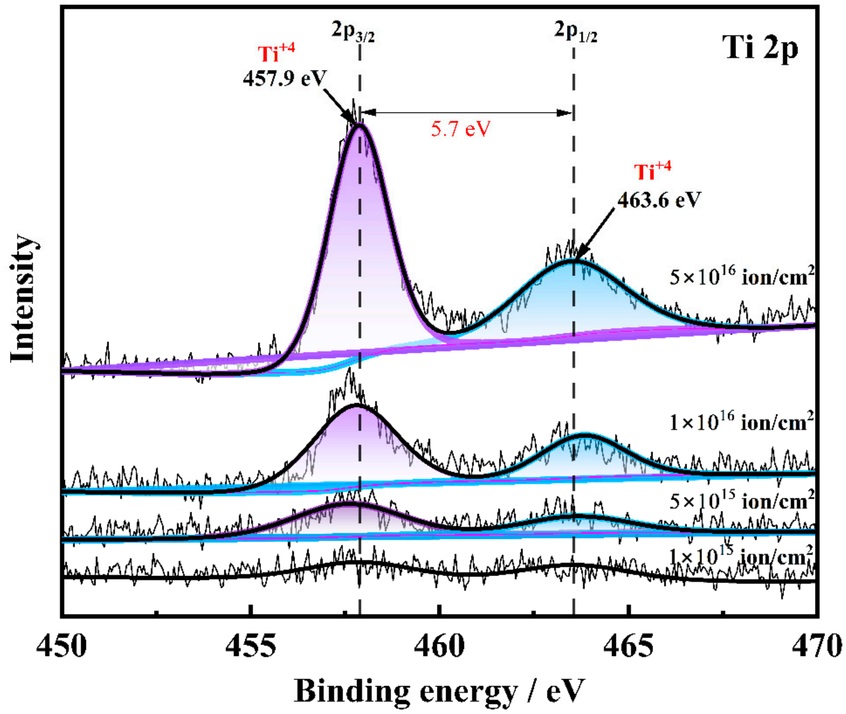

Figure S1. High-resolution XPS Ti 2p spectra of CuO NWs after Ti ion bombardment with different ion fluences.
